# GJA5 and ATP1A1 perturbations recapitulate inflammation-related beat irregularities in iPSC-based atrial myocardium tissue model

**DOI:** 10.3389/fimmu.2025.1719392

**Published:** 2026-03-06

**Authors:** Thomas Hutschalik, Albert Dasí, Leto L. Riebel, Maury Wiendels, Frederikus Bakker, Lucas J. A. M. Beckers, Koen C. Kriege, Susanne M. Valster, Roland C. M. Vulders, Ozan Özgül, Rémi Peyronnet, Blanca Rodriguez, Mariana Argenziano, Ulrich Schotten, Elena Matsa

**Affiliations:** 1 Ncardia Services B.V., Leiden, Netherlands; 2 Department of Physiology, Cardiovascular Research Institute Maastricht, Maastricht, Netherlands; 3 Department of Computer Science, University of Oxford, Oxford, United Kingdom; 4 Anatomy and Embryology, Leiden University Medical Center (LUMC), Leiden, Netherlands; 5 Department of Digital Standardization and Licensing Research, Intellectual Property and Standards, Royal Philips, Eindhoven, Netherlands; 6 Institute for Experimental Cardiovascular Medicine, University Heart Center Freiburg Bad Krozingen, and Faculty of Medicine, University of Freiburg, Freiburg, Germany; 7 Department of Cardiology, Maastricht University Medical Center, Maastricht, Netherlands; 8 Cellistic, Mont-Saint-Guibert, Belgium; 9 School of Biochemistry and Cell Biology, University College Cork, Cork, Ireland; 10 National Institute for Bioprocessing Research and Training, Dublin, Ireland

**Keywords:** atrial fibrillation, engineered 3D tissue, inflammation, IPSC, macrophage

## Abstract

Atrial fibrillation (AF) is the most common cardiac arrhythmia, linked to greater risk of heart failure, stroke and death. Inflammation has been connected to AF emergence, however mechanisms of inflammation-caused AF remain thus far elusive, leading to a lack of mechanism-based treatments. An isogenic, 3D tissue model containing hiPSC-derived atrial-like cardiomyocytes (aCM), cardiac fibroblasts (cfb), and cardiac macrophages was engineered using custom injection-molded pillar devices. Electrophysiological changes were examined via sharp electrode recordings, calcium imaging, and multi-electrode assays. Gene function was interrogated using siRNA knock-down, lentiviral overexpression, and pharmacological modulation. In silico tissue and whole-heart models validated findings under simulated stress and heterogeneous conditions. Activation of M1 macrophages led to a 50% reduction in contraction amplitude, action potential spike amplitude (aCM+cfb+M1: 61.3 mV ±13.9 *vs* control: 71.6 mV ±14.5, p < 0.01) and increased beat irregularity (M1: 150.7% ± 388.9 *vs* control, p < 0.001). Calcium transient amplitude was reduced (12.3 a.u. ± 14.7, p < 0.05) and upstroke velocity slowed. SCN5A knock-down reduced contraction amplitude (−51.9% ± 37.2, p < 0.01) without inducing arrhythmias, whereas combined GJA5 and ATP1A1 knock-down induced significant irregularity (403% ± 371.3, p < 0.001), increased conduction heterogeneity (+18%), and reduced velocity (−52.4%). In silico modeling confirmed that paired 50% downregulation of sodium-potassium pump and tissue conductivity induced AF under tachycardia even without ectopic activity. This work reveals a novel, inflammation-driven mechanism for AF initiation. Combined downregulation of GJA5 (connexin 40) and ATP1A1 (NaK ATPase) disrupted intercellular connectivity and ion flux, establishing a substrate for arrhythmogenesis. These results were robust across *in vitro*, genetic/pharmacological, and in silico models, defining new avenues for translational intervention.

## Introduction

Inflammation is known to be strongly associated with cardiovascular diseases, including cardiac arrhythmia (1, 2). For the most common arrhythmia, atrial fibrillation (AF), studies have shown a significant increase in inflammation markers and pro-inflammatory macrophages in AF patients and animal models (3–5). Inflammation has been proposed as a trigger of AF (6), and macrophages (Mφ) are considered as possible instigators of inflammation-caused AF, including by our group (7, 8).

Besides its pro-arrhythmic effect, inflammation is known to correlate with other cardiac dysfunctions. For example, cardiac contraction is reduced during cardiac inflammation, such as sepsis (9), and more recently a connection between Il-1β and reduced cardiac lymphatic muscle contraction has been proposed (10). How immune cell-related inflammation affects cardiac contraction has not yet been established.

hiPSC based models have already found wide use in disease modelling, with 3D cardiac models gaining increased prominence (11), including for AF modeling (12) and studying immune cell effects on heart failure (13). A 3D cardiac model incorporating non-inflammatory Mφ macrophages was also recently published, showing increase in contraction forces with Mφ included within tissues (14).

Mφ have been shown by our group to increase beat irregularity and reduce electrogram amplitude in atrial cardiomyocytes (aCM), after activation to a pro-inflammatory M1 subtype in human induced pluripotent stem cell (hiPSC) coculture models (8). Gene expression changes, for example upregulation of major histocompatibility complex (MHC) related genes and downregulation of electrophysiological genes (*GJA5*, *SCN5A*, *RRAD*, etc.), were identified and correlated with AF patient RNA (15). Nevertheless, a clear mechanistic link between observed transcription changes and emerging arrhythmias has not been shown.

This study sought to identify mechanistic relationships between activated M1 macrophage-induced inflammation and cardiac dysfunction, including arrhythmia initiation and contraction changes, using an isogenic 3D hiPSC atrial myocardium model, consisting of aCM, macrophages, and cardiac fibroblasts.

In this tissue model, macrophage activation led to reproducible arrhythmia emergence with concomitant reduction in tissue contraction. Sharp electrode recordings identified electrophysiological changes on a cellular level, including loss of sodium influx and increased action potential duration variability. Potentially causative target genes were screened using overexpression and knock-down methods, identifying that *SCN5A* knock-down resulted in lower contraction without arrhythmia, while combination of GJA5 and ATP1A1 loss was necessary for emergence of irregularity in beating intervals. *In silico* modeling of hiPSC confirmed tissue conductivity and Na+/K+ pump activity, primarily carried out by the two genes above, as causative of irregularity. A human *in silico* whole-atria model further showed the identified mechanism to cause AF even in the absence of ectopic activity, thus validating a mechanism for inflammation-induced AF.

## Methods

### Data availability

The data of this study are available from the corresponding author upon reasonable request.

### hiPSC culture

NC-030 hiPSC line (adult, episomal reprogramming, female, from renal epithelial cells, LUMC hiPSC core facility), NC-059 hiPSC line (fetal, episomal reprogramming, male, from CD34+ cord blood cells, NIH Center for Regenerative Medicine (CRM), CRMi001-A) and NC-196 (female, blood isolated PBMCs, episomal reprogramming, Maastricht University) were used. hiPSCs were cultured in mTeSR1 (StemCell Technologies) with Penicillin/Streptomycin (50 U/mL), seeded on Matrigel (Corning), and passaged twice weekly, including a DPBS- (Life Technologies) wash, using Accutase (Sigma-Aldrich) and medium supplemented with Fasudil (5 µM, LC Laboratories).

### Differentiation of hiPSC-derived atrial-like cardiomyocytes and culture

aCM differentiation protocols were previously described (8). Briefly, aCM were differentiated from NC-030, NC-059 and NC-196 hiPSC lines in monolayer, with 74k cells per cm^2^ seeded at day -1 on Matrigel (Corning) (1:100) before differentiation using Fasudil (5 µM) supplemented mTesR1. Medium was switched at day 0 to cardiac differentiation medium (Ncardia), inducing cardiac mesoderm by selectively activating and inhibiting Wnt pathways. Retinoic acid (RA) was used to induce atrial subtype. Medium change was performed every 2–3 days, and cells were dissociated and cryopreserved at day 14 using TrypLE Select (1x) (Life Technologies) in cardiac cryopreservation medium (Ncardia) with 10% DMSO (Sigma-Aldrich).

Cryopreserved aCM vials were thawed in Pluricyte culture medium (PCM, Ncardia) supplemented with Y27632 (10 µM, Axon Medchem) and cultured in PCM. aCM were seeded on Fibronectin (Sigma-Aldrich) diluted prior in DPBS+ (1:100, Life Technologies). Medium change was performed every 2–3 days.

### Monocyte differentiation, culture, and maturation to macrophages

hiPSC differentiation protocol for tissue-resident monocytes was adapted from Gutbier et al. (16) for all 3 lines. Dissociated hiPSC were transferred to AggreWell 800 wells (STEMCELL Technologies), aggregating into spheroids and cultured from day 0 to day 4 in mTesR1 with supplemented factors BMP4 (50 ng/mL, R&D systems), VEGF (50 ng/mL, R&D systems), and SCF (20 ng/mL, Miltenyi Biotec). Spheroids were moved at day 4 to Matrigel coated (1:100), 75 cm^2^) cell culture flasks (Corning). Medium was changed weekly thereafter with supplemented monocyte medium (X-Vivo 15 (Lonza), 50 U/mL Penicillin/Streptomycin, 0.05 mM 2-Mercaptoethanol (Gibco), 1% v/v GlutaMAX Supplement (Gibco), M-CSF (100 ng/mL, Gibco) and IL-3 (25 ng/mL, Gibco). Monocytes in suspension were harvested weekly from supernatants, after week 4 of differentiation.

Monocyte maturation to M1 macrophages was performed by adding 20 ng/mL GM-CSF (Gibco) to the culture medium for 6 days. Medium was refreshed with newly added GM-CSF at d3. Subsequently matured Mφ were activated through addition of 50 ng/mL LPS (InvivoGen) and 100 ng/mL IFN-γ (Peprotech) at d6 for 20h. Medium was changed thereafter, with fresh medium supplemented with 100 ng/mL LPS added to the cells for 4h.

### Cardiac fibroblast differentiation

Cfb were differentiated from NC-030, NC-059 and NC-196 by seeding 120,000 cells per wells of a 12 well plate coated with Matrigel (1:100), at d-3. mTeSR was used for daily medium changes up to d0. Atrial differentiation protocol was followed until d5. Medium was then switched to complete FibroLife serum-free fibroblast medium (CellSystems) with medium changes every 2 days. Wells were passaged at d8 using TrypLE Select (1x) onto Matrigel (1:100) coated 12 well plates. Cells were kept in culture until d14 and passaged onto uncoated 12 well plates. Cfb were cultured until they reached full confluence on d21 and were cryopreserved in FibroLife medium supplemented with 10% FBS (Gibco) and 10% DMSO.

### Cell fixation

Adherent cell cultures and 3D tissues were washed once with DPBS, then fixated using 4% paraformaldehyde (PFA) for 15 min at room temperature (RT) and subsequently rinsed twice more with DPBS. Cells in suspension were fixed according to the manufacturer’s protocol using Inside Stain Fix kit (Miltenyi Biotec). Fixations for nuclear transcription factors (COUP-TF II) were performed according to the manufacturer’s protocol using Stain Buffer and Transcription Factor Buffer Set (both BD Pharmingen).

### Flow cytometry

For flow cytometry a Novocyte Flow Cytometer 200 (ACEA Biosciences) was used. Washing steps and dilutions were performed with FACS buffer (Ncardia). Fixed aCM in suspension were co-stained with MLC2a REAfinity™ conjugated with APC (1:10, Miltenyi Biotec) and cTnT REAfinity™ conjugated with FITC (1:10, Miltenyi Biotec) antibodies and incubated for 15 min at RT. 100,000 cells were used per condition and samples were acquired at d14 and d28 of differentiation. Conditions were gated based on isotype controls (REA control APC, REA control FITC, Miltenyi Biotec). aCM were co-stained for COUP-TF II (primary antibody: 1:100, R&D Systems) and cTnT. aCM were incubated for 45 min at 4 °C with the primary COUP-TF II antibody in the dark. Cells were incubated thereafter with secondary antibody (APC) AffiniPure F(ab’)_2_ Fragment Donkey Anti-Mouse IgG (H+L), 1:500, Jackson ImmunoResearch), as well as the conjugated cTnT antibody for 45 min at 4 °C in the dark. Conditions were gated to isotype controls (REA control FITC, Purified Mouse IgG2a, κ, (BioLegend)).

Flow cytometry of monocytes was performed using live cells harvested from supernatant during differentiation (>d31). FACS Buffer was used for washes and dilutions. 100,000 cells were used per condition and triple stained with conjugated mouse anti-human, IgG1 antibodies for CD11b (1:20, APC), CD45 (1:20, PE), and CD14 (1:20, FITC, all BioLegend). Samples were incubated at 4°C in darkness for 20 min. Conditions were gated to isotype controls (Mouse IgG1-APC; PE; FITC, all BioLegend).

### RNA extraction, cDNA synthesis and qPCR

RNA extraction was performed using the NucleoSpin RNA Mini kit (Machery-Nagel) and cDNA synthesis using the iScript™ cDNA Synthesis Kit (BioRad) according to manufacturers’ protocols. qPCR was performed with an iQ5 thermal cycler (BioRad). using SsoAdvanced Universal SYBR^®^ Green Supermix (BioRad) according to manufacturer’s instructions. Primers are listed in **Supplementary Table S2**. Fold change was normalized to reference conditions and a housekeeping gene (GAPDH, ΔΔ), unless stated otherwise. For lentivirus transduced or siRNA treated aCM, Luna Cell Ready One-Step RT-qPCR Kit (New England Biolabs Inc.) was used, and gene expression was analyzed using a CFX Opus 384 Real-Time PCR System (BioRad), with expression normalized to housekeeping genes (GAPDH, ACTB) and reference conditions (ΔΔ).

### MEA seeding and recording

20.000 cells were droplet-seeded on fibronectin-coated (1:20 in DPBS+) MEA plates (Axion BioSystems). Cell culture medium was added after 2 hours for cells to adhere. A Maestro Pro (Axion BioSystems) was used for recordings set at 37 °C and 5% CO_2_. Plates were equilibrated for 30 min prior to 10 min recordings. Recording and processing was done using Axis Navigator (Axion BioSystems). Beat irregularity of recordings was calculated as described in Equation 1.

Equation 1: *Formula to calculate beat irregularity (%)*.


$$Beat\;irregularity\;\left(\%\right)=\frac{Standard\;Deviation\;of\;beat\;rate}{Mean\;of\;beat\;rate}\times 100$$

Isogenic cocultures of aCM and Mφ were combined prior to seeding in suspension and droplet-seeded, containing 20,000 and 5,000 cells, respectively per well, on 96 well Cytoview MEA plates (Axion BioSystems). PCM (200 µL per well) was used as culture medium, with M1 maturation/activation performed as previously described. For coculture characterization, 3 conditions were recorded at d8 (24h after activation): aCM+Mϕ, aCM+activation factors, aCM+M1.

### Heterogeneity mapping

Conduction direction vectors were obtained from siRNA-treated, aCM MEA recordings. These were defined as unit normalized vectors on each electrode, for an individual beat. A previously defined finite difference technique was used to calculate conduction velocities (17). Vectors were used to calculate heterogeneity as previously described (8). Heterogeneity is presented in arbitrary units (a.u.) on a scale of 0 to 1, with 0 being no heterogeneity between conduction vectors and 1 being absolute heterogeneity between vectors.

### Lentivirus overexpression

For *RRAD* overexpression, aCM monocultures were seeded and cultured on 96 well MEA plates as previously described. At day 6 post-seeding, aCM were co-transduced with either an *RRAD* insert lentiviral vector (pLV[Exp]-Puro-TRE>hRRAD[NM_001128850.2]) or *GFP* control vector, and a doxycycline-inducible TET lentiviral vector (pLV[Exp]-Bsd-EF1A>rtTA, all VectorBuilder), at a multiplicity of infection (MOI, e.g. vector per cell) of 5 for each vector, in 50 µl PCM per well. Cells were incubated overnight, and medium was replaced with PCM supplemented with either 0.1% DMSO (vehicle) or 10, 100, 1000 ng/ml doxycycline. Plates were recorded at d3 after transduction using MEA, and cells dissociated for qPCR analysis thereafter.

### Mold production

Pillar devices were designed and produced by Royal Philips for this study, using injection moldable silicone Elastosil LR 3040/40, on an Arburg injection molder (Arburg Allrounder 270 U 400–30 U) using regular injection molding settings. The final outer diameter of the pillar device was 6 mm. After sterilization via steam autoclaving, products were aseptically placed on the bottom of the wells of a 96-well cell plate.

### Engineered heart tissue formation

EHT formation protocol was based on Dostanić et al. (11) aCM were precultured prior to EHT seeding for 3 days on a fibronectin (1:100) coated 12 well plate at 1 million cells per well, in PCM. Cfb (passage 3-7) were precultured for 3 days on uncoated 12-well plates, at 350,000 cells per well, in Fibrolife medium. Mφ harvested from T75 differentiation flasks were moved to uncoated T75 flasks and cultured for 1 week prior to EHT seeding. On the day of EHT seeding, Mφ were removed from flasks by washing with macrophage culture medium, counted, centrifuged at 450 g for 5 min, resuspended in X-Vivo15 medium and placed on ice. Subsequently, cultured cfb were washed with PBS, dissociated using prewarmed (37 °C) TrypLE Express (1x) for 2 min and diluted in Fibrolife medium. Cells were counted, centrifuged at 250 g for 3 min, resuspended in FibroLife medium and placed on ice. Cardiomyocytes were dissociated using multi tissue dissociation kit 3 (Miltenyi Biotec) according to the manufacturer’s instructions. Cell dissociation solution was inactivated in PCM, and cells were counted and centrifuged at 250 g for 3 min. Cells were resuspended in PCM before being placed on ice. For EHT assembly, cells were mixed at a ratio of 56% aCM, 24% cfb, 20% Mφ. Tissues without Mφ had 70% aCM and 30% cfb. Cell suspensions were combined at 39,200 aCM, 16,800 cfb and 14,000 Mϕ per EHT (70,000 cells total, per EHT). Combined suspensions were centrifuged at 450 g for 5 min and resuspended in 1.42 µl formation medium per well. Formation medium consisted of MEM α (Gibco) supplemented with Penicillin/Streptomycin (10U/µl, 1:100), L-AA-2-phosphate (200 µM, ThermoFisher Scientific) and FBS (1:10). ECM solution was prepared in parallel, containing 1.46 µl Collagen 1 solution (TeloCol^®^-3, Advanced Biomatrix), 0.18 µl 10x DMEM (Merck), 0.22 µl 0.1M NaOH and 0.32 µl Matrigel per well. ECM solution was gently mixed, cell suspension was added to the prepared solution, gently mixed and placed on ice. The culture plate with pillar devices was placed on ice and 3.6 µl of final cell suspension was added to each pillar device, covering the complete inlay surrounding the pillars. Unused wells were filled with sterile, tissue culture grade water (Gibco). The plate was then incubated at 37 °C and 5% CO2 for 20 minutes, until the cell mixture became opaque. 200 µl of formation medium supplemented with 5 ng/ml FGF (Miltenyi Biotec) (and 20 ng/ml GM-CSF for M1 conditions) was slowly added to each EHT well. Medium was changed at d3, using PCM with same supplements. M1 activation for EHT was performed as previously described. At time of EHT formation, aCM were at 24 days post differentiation initiation.

### Tissue contraction analysis

Brightfield video recordings of tissue were recorded at 100 fps and analyzed using MUSCLEMOTION software (18). Contraction amplitudes were normalized to aCM+cfb EHT conditions. Beat irregularity was calculated as previously described (Equation 1) and normalized to aCM+cfb EHT conditions.

### Calcium imaging

Calcium imaging was performed using an FDSS/μCELL Kinetic Plate Imager (Hamamatsu). Calcium was labelled using FLIPR^®^ Calcium 6 assay kit (Molecular Devices) according to manufacturer protocol. Calcium recordings were analyzed using FDSS/μCELL Kinetic Plate Imager software.

### Intracellular action potential (sharp electrode) recordings

For 2D aCM, cells were seeded on coverslips (ThermoFisher Scientific) and recorded at 7 ± 2 days after seeding in a perfusion chamber (RC-26G, Warner Instruments). 3D tissues were seeded in 35 mm dishes with pillar molds (custom, Royal Philips) and placed in a perfusion chamber after 7–8 days of seeding. Cells were placed in a bath solution with constant flow from a peristaltic pump (Easy-Load II Pump, Masterflex L/S) with a flow rate of 2 mL/min heated to 35 ± 2 C. Bath solution for 2D cells consisted of a modified, oxygenated, normal Tyrode’s solution (KCl 5.5 mM, NaCl 140 mM, MgCl2–1 mM, HEPES 10 mM, CaCl2 1.8 mM, Glucose 10 mM), tissues were perfused with PCM. Solutions were heated with a chamber heater (PH1, Warner Instruments), using a flow-through (SH-27B, Warner Instruments) and two-channel controller (TC-344B; Warner Instruments). Cells were penetrated with pulled glass capillaries (Clark borosilicate with filament, OD 1.00/ID 0.58, 100 mm, Warner Instruments) with microelectrodes, resulting in a resistance of 15–20 MΩ. Capillaries were pulled using a Sutter P-97 micropipette puller (Sutter Instrument). Capillaries were filled with a solution containing 3 M KCl and were connected to a bridge amplifier (BA-01X, NPI Electronic) using an Ag-Ag-Cl electrode, with an Ag-AgCl reference electrode (E205, Ø 1.0 mm, Harvard Apparatus). The electrode was moved using a TSC Sensapex micromanipulator (Oulu, Finland), which was controlled by an IX70 microscope (Olympus). Action potentials were recorded at 50 kHz with a 10 kHz filter using a LabView (National Instruments) script (custom).

### Brightfield imaging

Brightfield imaging of fibroblasts was performed using a ToupCam LCMOS05100KPA Camera (ToupTek) and a Nikon Eclipse TS100 microscope (Nikon). Unless stated otherwise, tissues were recorded using a USB 3.0 digital camera UI-33240CP-C-HZ-TL (Thorlabs) mounted on an Eclipse Ti microscope (Nikon), with tissues placed in a custom-build environmental chamber at 37 °C and 5% CO2. Pillar devices were evaluated by either using a digital microscope (Keyence) or with an inverted microscope outfitted with a camera (Leica, DM IL LED).

### Immunofluorescence staining

ImageXpress Micro Confocal high content imager (Molecular Devices) was used to image PFA fixed adherent cells seeded on black 96 well µClear^®^ CELLSTAR^®^ plates (Greiner) or 3D tissues removed from pillar molds and placed in a 384 well µClear^®^ CELLSTAR^®^ plates (Greiner). Dilutions used PBST-FBS (DPBS, Life Technologies; FBS, Gibco; Tween20, ThermoFisher Scientific). Samples were blocked with PBST-FBS and washed with PBST.

Fibroblasts and Ncyte ^®^ vascular smooth muscle cells (Ncardia) were incubated with primary antibodies (Vimentin, anti-human, human, FITC (1:50, Miltenyi)), Alpha smooth muscle actin [1a4] mab1420 mouse (1:50, R&D Systems) or NG2/MCSP PE mouse ab (1:100, R&D Systems) overnight at 4 °C on a plate shaker. Samples were incubated with secondary antibodies (IgG (H+L) Alexa Fluor^®^ 488 goat anti human (1:500, ThermoFisher Scientific), Goat anti-Mouse IgG2b, Alexa Fluor^®^ 647 conjugated (1:200, ThermoFisher Scientific)) for 1h at RT, before being washed and imaged.

Tissues were incubated with primary antibodies (cTnT REAfinity conjugated FITC (1:100, Miltenyi Biotec)) and CX3CR1 rabbit anti-human (1H14L7) (1:250, Invitrogen) or Collagen I Polyclonal Antibody (1:100, ThermoFisher Scientific) overnight at 4 °C on a plate shaker. Samples were incubated with secondary antibodies (IgG (H+L) Alexa Fluor^®^ 488 goat anti human (1:200, ThermoFisher Scientific), IgG (H+L) Alexa Fluor^®^ 594 Donkey anti Rabbit (1:200, ThermoFisher Scientific), Donkey Anti-Rabbit IgG (H+L) antibody Alexa Fluor^®^ 488 (1:200, ThermoFisher Scientific) and DAPI (1:1000, Invitrogen) for 1h at RT, before being washed and imaged. Collagen deposition was quantified using a custom protocol in ImageXpress MetaXpress (Molecular Devices), which analyzed the integrated fluorescence intensity of whole tissues divided by the whole tissue area.

### Compound treatment

Hydrocortisone (10 µM) and vehicle (0.1% DMSO) were added to EHTs. Tissues were seeded and cultured as previously described and compound was added during all medium changes during maturation and activation as described prior, including at d6, d6 + 20h, d7 and d8 after seeding. Video recordings were performed on d8 after seeding.

MEA recordings of 2D aCM monocultures and aCM+Mφ cocultures were treated with flecainide (0.01, 0.1, 1, 10 µM), digoxin (0.1, 1, 10, 100 nM) and 4-aminopyridine (4-AP; 0.05, 0.5, 5, 50 µM; all Selleckchem) individually or combined (vehicle; 0.1% DMSO). Serially diluted compounds were added to wells 2h prior to MEA recordings. Wells showing cessation of beating were excluded from statistical testing.

### siRNA inhibition

For siRNA knock-down, 2D aCM monocultures were seeded and recorded on MEA plates as previously described. After 6 days of culture, siRNA (Silencer™ Select siRNA, ThermoFisher Scientific) was added according to manufacturer’s instructions, using 0.3 µl Lipofectamine^®^ RNAiMAX (ThermoFisher Scientific) in 50 µl optiMEM (Gibco) per well as cationic lipid transfection agent, with 150 µl PCM added per well afterwards. Final siRNA concentration was 5 pmol. Silencer™ Select No. 1 siRNA (ThermoFisher Scientific) was used as negative control. Combined siRNA treatments were performed additively, with matching negative control concentrations emulating increased target siRNA concentrations. MEA recordings were performed 5 days after siRNA addition. For 3D tissues, siRNA treatment was performed as described here, on precultured aCM only, 1 day prior to EHT formation.

### *In silico* hiPSC-CM tissue

The virtual tissue of hiPSC-CMs was created using the Paci et al. (19) cellular model. The tissue was composed of 10,000 elements of 250 μm edge length, resulting in a square mesh of 6.25 cm (2). To capture beating irregularity, the spontaneous activity of hiPSC-CMs was simulated for 50 seconds. For every element of the mesh, an activation was registered when the cellular depolarization reached 0 mV, and irregularity was then calculated as described by Equation 1.

Different tissue configurations were created to reproduce the spatial heterogeneity of the inflammatory response (**Supplementary Figure S6**). Firstly, a control tissue was developed by assigning baseline properties to all hiPSC-CMs (i.e., no remodeling). Then, four additional tissues were built to cover variability in spatially heterogeneous remodeling. In all four scenarios, the electrophysiological variations were applied in half of the cells, while the other half preserved the hiPSC-CM control properties. To capture the spatial heterogeneity of the inflammatory response, the electrophysiological remodeling in the tissue was applied in circular patches, so that both remodeled and control hiPSC-CMs coexisted in the same environment.

In all four remodeled tissues, six electrophysiological variations were investigated: individual downregulation of I_Na_, I_NaK_ and tissue conductivity (calibrated to achieve a conduction velocity of 45 cm/s in control), as well as the combined effect paired downregulation. These electrophysiological parameters were reduced either by 25% or 50% with respect to their control values (**Supplementary Figure S6**).

### *In silico* human whole-organ atria

Whole-organ simulations were conducted using the detailed 3D human atrial model described in Ferrer et al. (20), which considers 515,005 elements of 300 μm edge length. The Courtemanche et al. (21), cellular model was used to describe the electrophysiology of the atria, by including its single-cell properties in the left atrial tissue and scaling them in other atrial regions, as described previously (22). Regional heterogeneities in conduction velocity and anisotropy ratio were also considered (22), setting the longitudinal velocity in the atrial body to 0.8 m/s.

To model the effects of inflammation-induced electrophysiological remodeling, circular patches with a radius comparable to that in the hiPSC-CMs tissue (0.5 cm) were included in the atria. Patches were distributed in the left and/or right atria either localized in the venous portion or spread out across the whole atria. Combining control, localized or spread-out scenarios for the left and right atria resulted in eight configurations of spatially heterogeneous remodeling. Mesh elements circumscribed by these patches presented a 50% down-regulation of I_NaK_ and a 50% reduction in the longitudinal and transversal conductivity. A control mesh was also created, preserving the baseline properties in all atrial cells (i.e., no remodeling).

In all nine scenarios, sinus rhythm stimulation was applied for 10 beats at 85 beats per minute (i.e., cycle length of 700 ms). Subsequently, the pacing rate was increased to 190 beats per minute (i.e., cycle length of 310 ms) during 5 beats to simulate a situation of stress.

The multi-scale monodomain equation of the transmembrane voltage was solved using the high-performance open-source tool, MonoAlg3D (23).

### Statistical analysis

A *P* value ≤0.05 was designated as significant. All variance is given in standard deviation. Calculations were made using Excel (Microsoft). Statistical analysis and graph generation were performed using GraphPad Prism 8 (GraphPad Holdings, LLC). Independent experiments are denoted as ‘N’, individual samples (e.g., tissues, cells, wells) as ‘n’. For two group comparisons of normally distributed data, student-t test (paired or unpaired) was performed, while for non-normally distributed data, Mann-Whitney testing was used. For >2 group comparisons of normally distributed data, One Way ANOVA was used, and for non-normally distributed data, Kruskal-Wallis test was performed. For comparisons between multiple combined groups, nested t-tests and Two Way ANOVA were used.

## Results

### Atrial-like cardiomyocytes, cardiac tissue-resident macrophages, and cardiac fibroblasts derived from hiPSC show subtype specific phenotypes

Atrial-like cardiomyocytes (aCM) and Mϕ macrophages were derived from three hiPSC lines (NC-030, NC-059, NC-196) as previously described and characterized by our group (8). Differentiated cells for this study expressed high levels of lineage-specific markers (**Supplementary Figures S1A–D**). aCM expressed both cardiac marker cTnT and atrial marker COUP-TF II (double positive 75.3% ± 5.8). Mφ expressed leukocyte marker CD45, CD11b and monocyte/macrophage marker CD14 (94.7% ± 5.4; 94% ± 5.1; 70.3% ± 16.7 respectively. Cardiac fibroblasts (cfb) were differentiated from the same three donor hiPSC lines using a protocol optimized in this study, based on previously published information (24). Cfb showed cell type specific elongated, spindle-like morphology (**Supplementary Figure S1E**), gene expression (*COL1A1*, *MMP2;***Supplementary Figure S1F**) and structural protein expression (Vimentin; **Supplementary Figures S1G, H**). Markers of other mesenchymal cell subtypes such as CD31 and a-actin were not expressed in cfb, confirming the absence of endothelial and smooth muscle cells, respectively (**Supplementary Figures S1G, H**).

### Macrophages functionally integrate into 3D hiPSC-based tissue model of atrial myocardium

**Figure 1:**
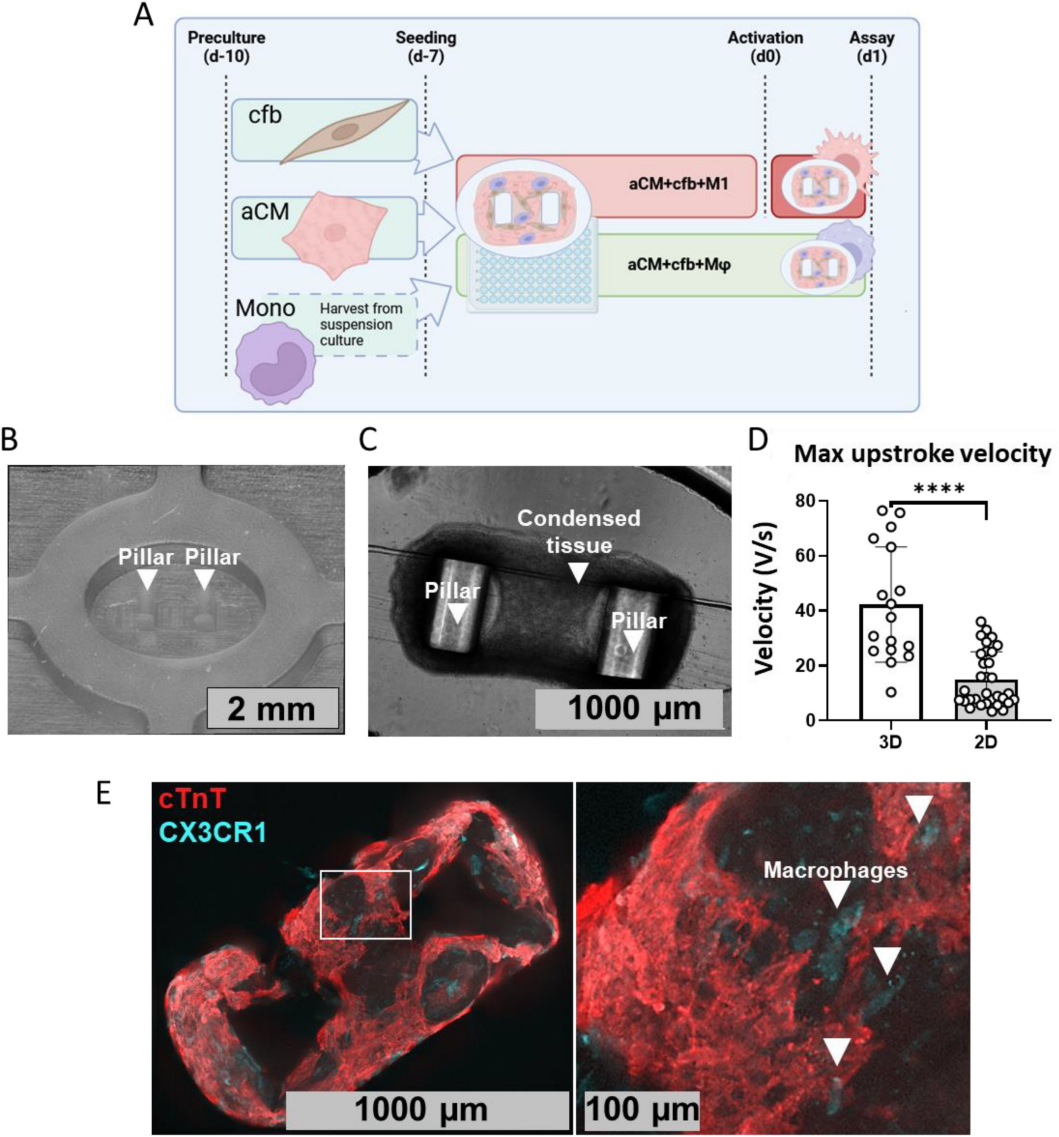
Fabrication of 3D condensed tissues incorporating M1 macrophages. **(A)** Schematic of 3D tissue formation protocol, consisting of aCM, cfb, and monocyte preculture for 3 days (d-10), tissue seeding in 96 well plates (d-7) subsequent culture and M1 maturation for 7 days (d-7 to d0) and activation of M1 (d0). **(B)** Brightfield image of the injection molded pillar device used for seeding tissues. **(C)** Annotated representative brightfield image of condensed 3D tissue consisting of aCM+cfb. 9**D)** Sharp electrode recordings of single cell aCMs in 3D tissues consisting of aCM+cfb (n=17/N=4) compared to 2D monolayer of aCM for (n=31/N=3), demonstrating significant increase in upstroke velocity for 3D tissues (Mann-Whitney test). **(E)** IF staining for cardiac marker cTnT and cardiac tissue resident macrophage marker CX3CR1 (26) in aCM+cfb+M1 tissue. Zoomed-in image showing area marked by white rectangle, white arrows indicate macrophages. ****P<0.0001.

A protocol for fabrication of a 3D hiPSC myocardium model was optimized in this study, incorporating activated pro-inflammatory M1 (**Figure 1A**). For this, aCM, monocytes and cfb were separately pre-cultured and seeded in 96 well plate-based pillar devices. Prototypes of molds and pillar devices were designed and produced in collaboration with Royal Philips, using silicone rubber, Elastosil^®^, and consisting of an oval shaped reservoir (3x2 mm) harboring two 1 mm pillars (**Figure 1B**). Injection molding is a well-known, reliable and reproducible production technique (25), which allows for manufacturing of pillar devices on a large scale. In this study, injection molding enabled reproducible production of pillar devices with consistent geometry and transparent materials, allowing for easy visual recording. Devices were compatible with brightfield imaging, fluorescent imaging and sharp electrode recording, as shown by results in this study.

**Figure 2:**
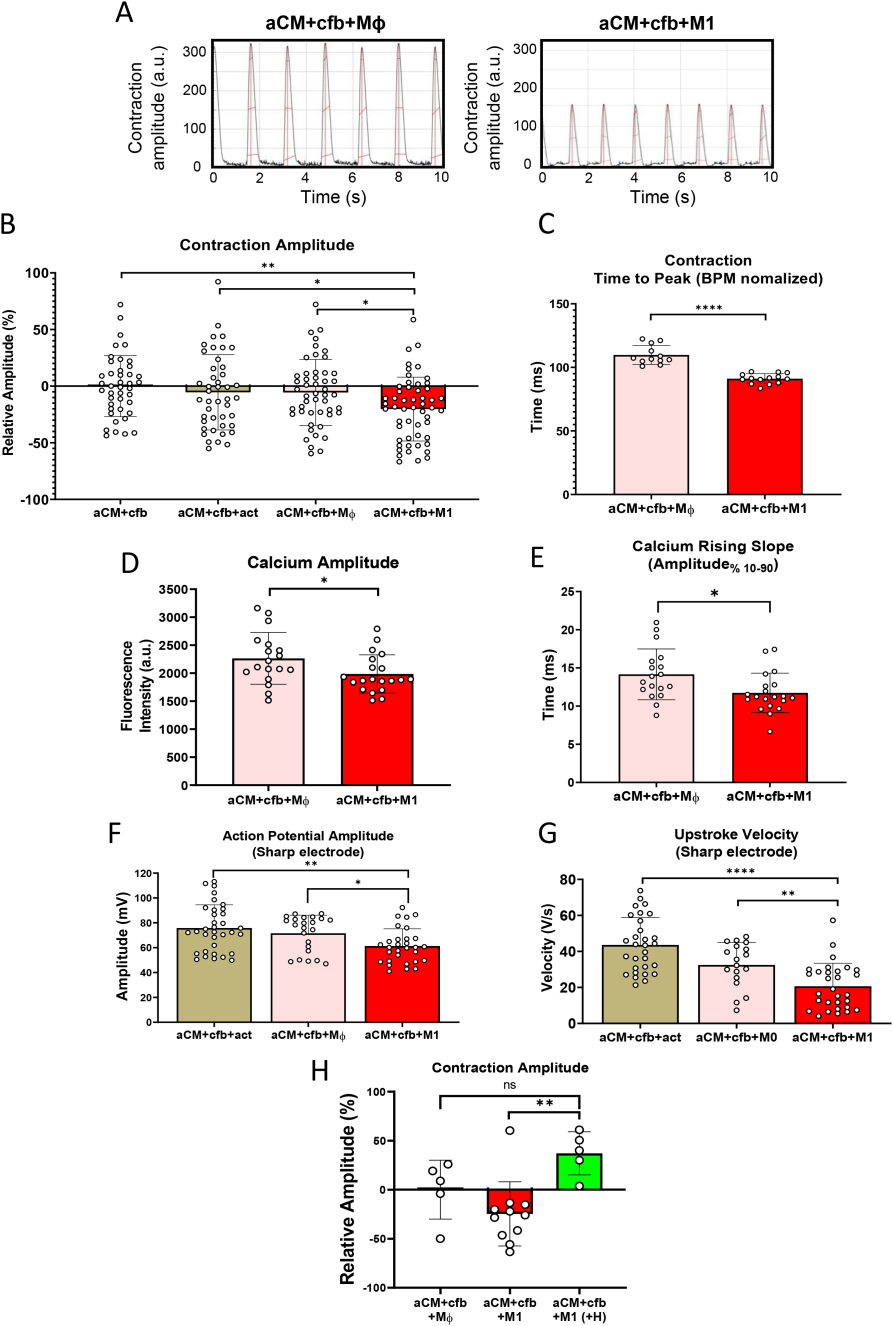
Activation of M1 macrophages in 3D tissues reduces contraction, calcium transient and sodium spike amplitude. **(A)** Representative contraction traces of tissue containing activated (M1) and non-activated (Mφ) macrophages. **(B)** Relative contraction amplitude changes of 3D tissues at d1 after activation, normalized to aCM+cfb (n=42,44,49,53/N=6 One Way ANOVA). **(C)** Contraction time to peak from baseline, normalized to BPM, indicating a shortened time to peak contraction in 3D tissues with M1 *vs*. Mφ (n=12,14/N=1, unpaired student t-test). **(D)** Calcium imaging analysis showing calcium amplitude in tissues, at d1 after activation and **(E)** rising slope times from 10 to 90% (n=18,20/N=3 unpaired student t-test). **(F)** Sharp electrode recordings of action potential amplitude (n=34,23,30/N = 4 One Way ANOVA) and **(G)** upstroke velocity in individual aCM within tissues, at d1 after activation (n=30,18,30/N = 4, One Way ANOVA).( **H)** Relative, video-based contraction amplitude changes in 3D tissues, at d1 after activation, including hydrocortisone treated tissues (+H, 10µM), normalized to aCM+cfb+Mφ (n=5,10,5/N=1 One Way ANOVA). ns: not significant, *P<0.05, **P<0.01, ****P<0.0001.

Cells condensed into contracting tissues aggregated around the pillars, during 24h post-seeding (**Figure 2C**). All used pillar devices showed deflection of pillars from cardiomyocyte contraction upon EHT formation. Sharp electrode recordings of aCM within tissues showed similar action potential amplitudes and maximum diastolic resting potentials compared to 2D cultured aCMs (**Supplementary Figure S2A**). Nevertheless, 3D aCM cultures showed significantly higher maximum upstroke velocities (42.28 V/s ± 21.04) compared to 2D ones (14.95 V/s ± 10.08). This result shows some model-dependent effect of electrophysiological maturation (**Figure 1D**).

Macrophages made up 20% of total cell count during seeding and integrated within tissues after formation. Immunofluorescence staining (IF) confirmed M1 were uniformly spread along the cardiac tissue and expressed cardiac tissue-resident marker, CX3CR1 (26) (**Figure 2E**). Tissues were stained for collagen 1 to assess extracellular matrix (ECM) deposition mainly from cfb. IF imaging showed collagen deposited along the tissues (**Supplementary Figure S2B**), indicating the expected functionality for fibroblasts. Collagen deposition was significantly lower (85 a.u. ± 15 *vs*. 118 a.u. ± 30, P<0.05) in tissues with activated M1 compared to tissues with non-activated Mϕ (**Supplementary Figure S2C**), consistent with previously published effects of M1 on ECM deposition (27) and suggesting the ability of M1 to be activated and functional within the 3D tissue structures. The result further suggests absence of M2 macrophages, as the subtype is known to increase fibrosis and collagen deposition in the ECM (28). Collagen deposition in M1 tissues reached levels closer to Mφ over a time period of 15 days post-activation but was overall significantly lower (P<0.01), (**Supplementary Figure S2D**), demonstrating a reduced effect over time after single M1 activation at d0.

Overall, this data presents the successful integration of M1 in viable 3D tissues using a custom-made, 96 well plate-based, injection molded pillar device to model subtype specific inflammation effects.

### Macrophage-mediated inflammation causes reduction of sodium spike, calcium amplitude and contraction amplitude in 3D tissues

To better understand the effects of incorporating M1 into aCM cardiac tissues, contraction videos of tissues formed in pillar devices were recorded and analyzed using MUSCLEMOTION software (18) (**Supplementary Figure S3A**, **Supplementary Video S1**). Data showed that activated M1 caused significantly lower contraction amplitudes compared to control conditions (14.69% ± 28.2 to aCM+cfb+Mφ, **Figures 2A, B**, and **Supplementary Figure S3B**). Further, time to contraction peak in 3D tissues with M1 was significantly shorter (17.1% ± 3.9, **Figure 2C**). The observations were further investigated using calcium imaging. In line with lower contraction amplitudes, amplitudes of calcium transients were lower in M1 tissues compared to Mφ (12.3% ± 14.7, **Figure 2D**). Additionally, calcium rising slopes were significantly shorter in the M1 condition (17.2% ± 17.7, **Figure 2E**). Area under the curve and calcium concentration decay slopes were not significantly affected by M1 (**Supplementary Figure S3C**).

Electrophysiological consequences of activated M1 were further investigated using the sharp electrode technique in 3D tissues (**Supplementary Figure S3D**). Action potential amplitude was significantly lower in M1 tissues (aCM+cfb+M1: 61.3 mV ± 13.9, aCM+cfb+Mϕ: 71.6 mV ± 14.5; **Figure 2F**). Tissues with M1 also showed significantly slower maximum upstroke velocity (aCM+cfb+M1: 20.7 V/s ± 12.8, aCM+cfb+Mϕ: 32.5 V/s ± 12.4; **Figure 2G**). Finally, reduction in electrogram amplitude was investigated in 2D cocultures of aCM and macrophages using MEA recordings; comparing monolayer cocultures of aCM and M1 (aCM+M1) to aCM with activation factors (aCM+act) and cocultures without activation (aCM+Mφ), M1 cocultures showed significantly lower electrogram amplitudes (NC-196: aCM+M1 1.08 mV ± 0.54; aCM+M0 1.54 mV ± 0.91; aCM+act 1.47 mV ± 0.56) (**Supplementary Figure S3E**), confirming prior results in 2D cultures (8).

To ascertain that the observed contraction reduction was caused by M1-induced inflammation, 3D tissues were treated with hydrocortisone, an immunosuppressant glucocorticoid inhibiting M1 activation, to suppress inflammation. Based on video contraction analysis, hydrocortisone treatment, at 10 µM, resulted in higher contraction amplitudes compared to untreated M1 tissues (82.1% ± 26.1 increase compared to aCM+cfb+M1, **Figure 2H**).

Overall, this data supports that in multi-cell type 3D atrial myocardium tissues, macrophage-induced inflammation leads to lower contraction and calcium amplitudes, as well as lowered action potential amplitude and upstroke velocity. This is in agreement to studies showing worsened atrial contraction in AF (29) and negative effects on sodium influx due to aCM-M1 co-culture in monolayer models (8).

### Downregulation of *SCN5A* results in lower contraction amplitude

**Figure 3:**
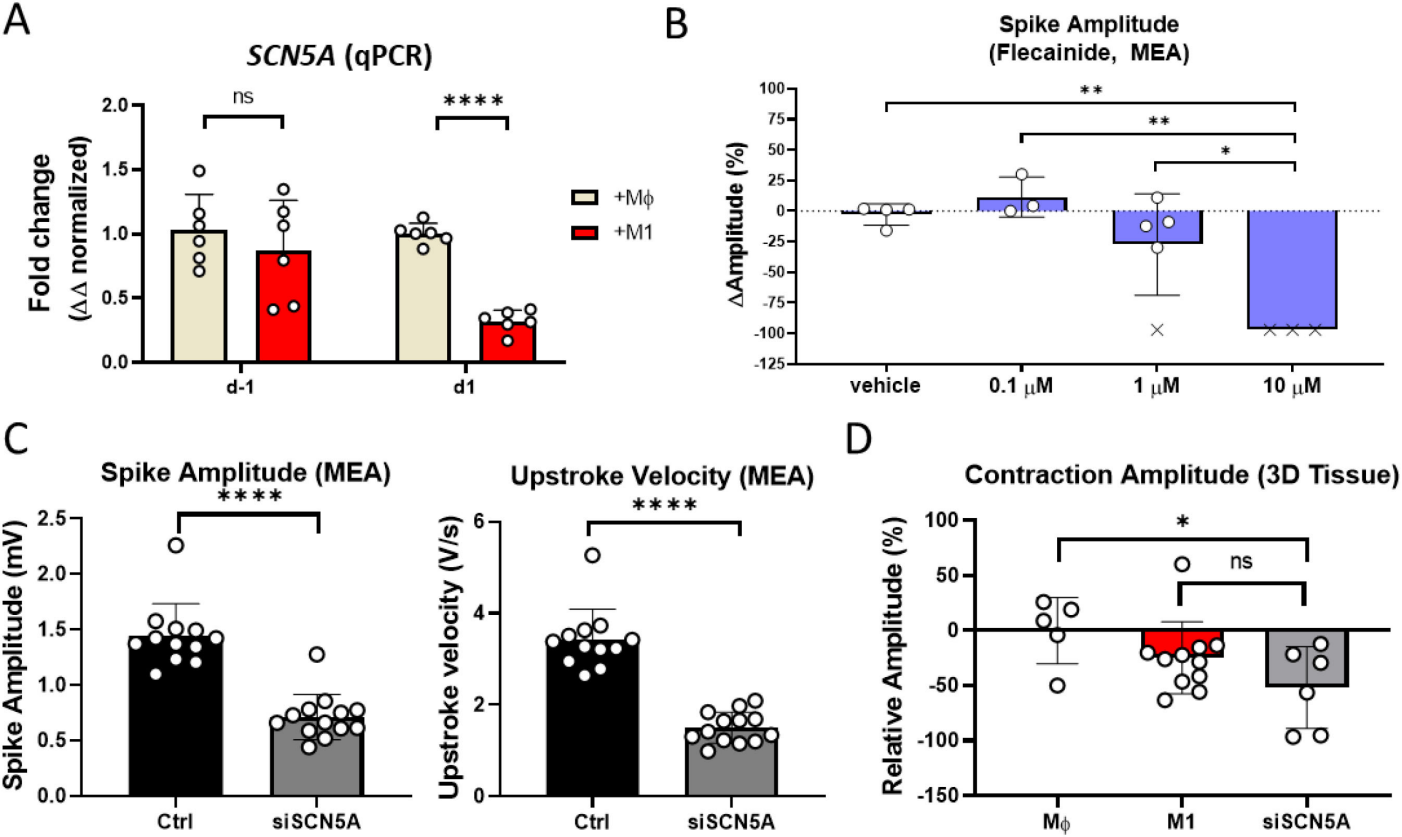
SCN5A downregulation resulted in reduced sodium spike and contraction amplitude. **(A)** qPCR expression of *SCN5A* in purified aCM after coculture with either M1 or Mϕ before (d-1) and after (d1) activation, normalized to GAPDH and Mϕ expression levels (n=6/N=2, unpaired student t-test). **(B)** Spike amplitude in aCM, at 0.5h after flecainide addition, recorded using MEA. (x) denote cessation of beating with non-detectable spikes (n=4,3,5,3/N=1, One Way ANOVA). **(C)** Spike amplitude and upstroke velocity in aCM treated with siRNA against *SCN5A* or control, recorded using MEA (n=12,13/N=3, unpaired student t-test). **(D)** Contraction amplitude based on video recordings in aCM+cfb 3D tissues with either Mϕ, M1, or Mϕ+siRNA(*SCN5A*), normalized to Mϕ, indicating a reduction in contraction amplitude through reduced *SCN5A* expression (n=5,11,6/N=2, One Way ANOVA). ns: not significant, *P<0.05, **P<0.01, ****P<0.0001.

Inflammation-induced lower spike amplitude and correlated reduction of *SCN5A* has been previously shown Click or tap here to enter text (8,). (30) Click or tap here to enter text. *SCN5A* downregulation was confirmed in this study using qPCR in aCM isolated from M1 or Mφ cocultures prior to (d-1) and after (d1) activation (68% ± 7.8, **Figure 3A**). To further investigate the correlation between *SCN5A*, sodium influx and contraction amplitude, aCM were treated with the Nav1.5 inhibitor, flecainide. Results indicated a reduction in spike amplitude, suggesting a correlation between the latter and sodium influx (**Figure 3B**). Next, siRNA inhibition of *SCN5A* was performed on aCM, with MEA recordings showing significant reduction in spike amplitude (-51% ± 14) and maximum sodium upstroke velocity (-56% ± 23, **Figure 3C**), recapitulating the cellular phenotype observed in aCM+cfb+M1 3D tissues. aCM+cfb+Mϕ tissues treated with *SCN5A* siRNA also showed significantly reduced contraction amplitude (-51.9% ± 37.16, **Figure 3D**).

In summary, the data suggests downregulation of *SCN5A* to be causative of lower contraction amplitude, pointing at inflammation-induced reduction of *SCN5A* as a potential pathophysiological mechanism.

### M1-induced inflammation led to arrhythmia-like phenotype

**Figure 4:**
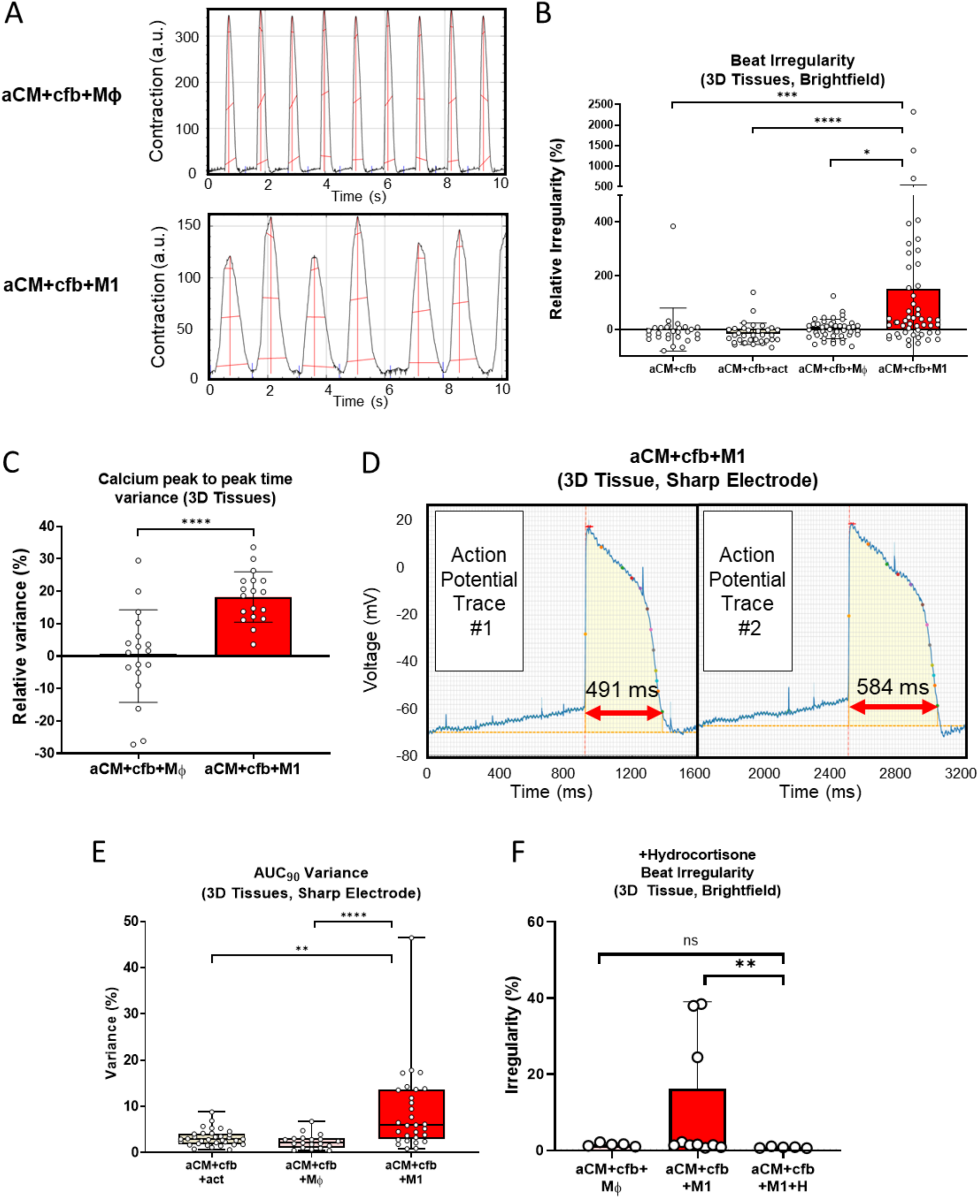
M1 activation resulted in irregular beating and electrophysiological perturbations of tissues. **(A)** Representative traces generated with MUSCLEMOTION software from video recordings of contracting tissues, demonstrating irregular beat patterns in tissues with M1. **(B)** Beat Irregularity in 3D tissues recorded using brightfield videos and analyzed with MUSCLEMOTION software (N = 28,34,45,51/N=6, Kruskal-Wallis test). **(C)** Peak to peak time variance in calcium transient peaks from 3D tissues, normalized to Mϕ control condition (n=18/N=3 unpaired student t-test). **(D)** Representative sharp electrode action potential traces of two consecutive aCM action potentials, showing an alternans pattern with variation in APDs and AUC_90_ in consecutive action potentials (arrows indicating APD_90_.**(E)** AUC_90_ variance of sharp electrode recorded action potentials from individual aCM in 3D tissues (n=33,19,29/N=4, Kruskal-Wallis test). **(F)** Beat irregularity in 3D tissues treated with 10 µM hydrocortisone, based on video recordings analyzed using MUSCLEMOTION software (n=5,11,5/N=2 Kruskal-Wallis test). ns: not significant, *P<0.05, **P<0.01, ***P<0.001, ****P<0.0001.

Video recordings of 3D tissues showed irregular contractions, including missed beats and fibrillation-like arrhythmia (**Figure 4A**, **Supplementary Figure S4A**, **Supplementary Video S2**). Video analysis using MUSCLEMOTION software revealed that M1 induced significantly higher beat irregularity compared to control aCM+cfb+Mφ (150.7% ± 388.9, **Figure 4B**). Irregularity was also observed using fluorescent dye-mediated calcium transient recordings, which showed significant increase in variance between calcium peaks (18.22% ± 7.76, **Figure 4C**). Beat irregularity was confirmed in 2D cocultures of aCM and M1 (**Supplementary Figure S4B**) using MEA recordings. 2D cocultures also presented lower conduction velocity in aCM+M1 conditions (25% ± 13 compared to Mφ, **Supplementary Figure S4B**). Next, direct cocultures of aCM and M1 (aCM+M1) were compared to aCM with activation factors (aCM+act) and cocultures without activation (aCM+Mϕ) using multi electrode assays (MEA). In these conditions, M1 significantly lowered conduction velocity (NC-196: aCM+M1 0.09 mm/ms ± 0.05; aCM+Mφ 0.12 mm/ms ± 0.07; aCM+act 0.12 mm/ms ± 0.04) and significantly increased beat rate irregularity (NC-196: aCM+M1 3.56% ± 12.11; aCM+M0 0.56% ± 0.4; aCM+act 0.61% ± 0.37).

Sharp electrode recordings of individual aCM within tissues revealed that M1-inflammation resulted in a less negative maximum diastolic potential (12.3% ± 2.5, **Supplementary Figure S4C**), and in alternans within tissues (**Figure 4D**). Further, sharp electrode recordings showed a significantly higher AP variance in M1 tissues for both AUC_90_ and APD_90_ compared to controls (M1: 8.9% ± 9, 6.2% ± 9.4 Mϕ: 2.4% ± 1.6, 1.1%± 1, respectively), pointing at electrophysiological perturbations on a cellular level (**Figure 4E**, **Supplementary Figure S4D**).

M1-induced irregularity was inhibited by suppressing M1 activation using hydrocortisone treatment, 10 µM, further pointing at M1 activation being causative of the irregularity (**Figure 4F**). This finding is congruent with meta-studies of clinical trials, which have shown anti-inflammatory drugs to reduce fibrillation (31) and the occurrence of post-operative AF (1, 32, 33). In summary, data from multiple functionality assays demonstrate M1 inflammation to increase beat irregularity of tissues and cause electrophysiological perturbations on a single cell level, suggesting that inflammation can be a potential cause of pro-arrhythmia, as observed *in vitro (*8) and in animal models (7, 30).

### Combined downregulation of GJA5 and ATP1A1 expression recapitulates pro-arrhythmic phenotype

**Figure 5:**
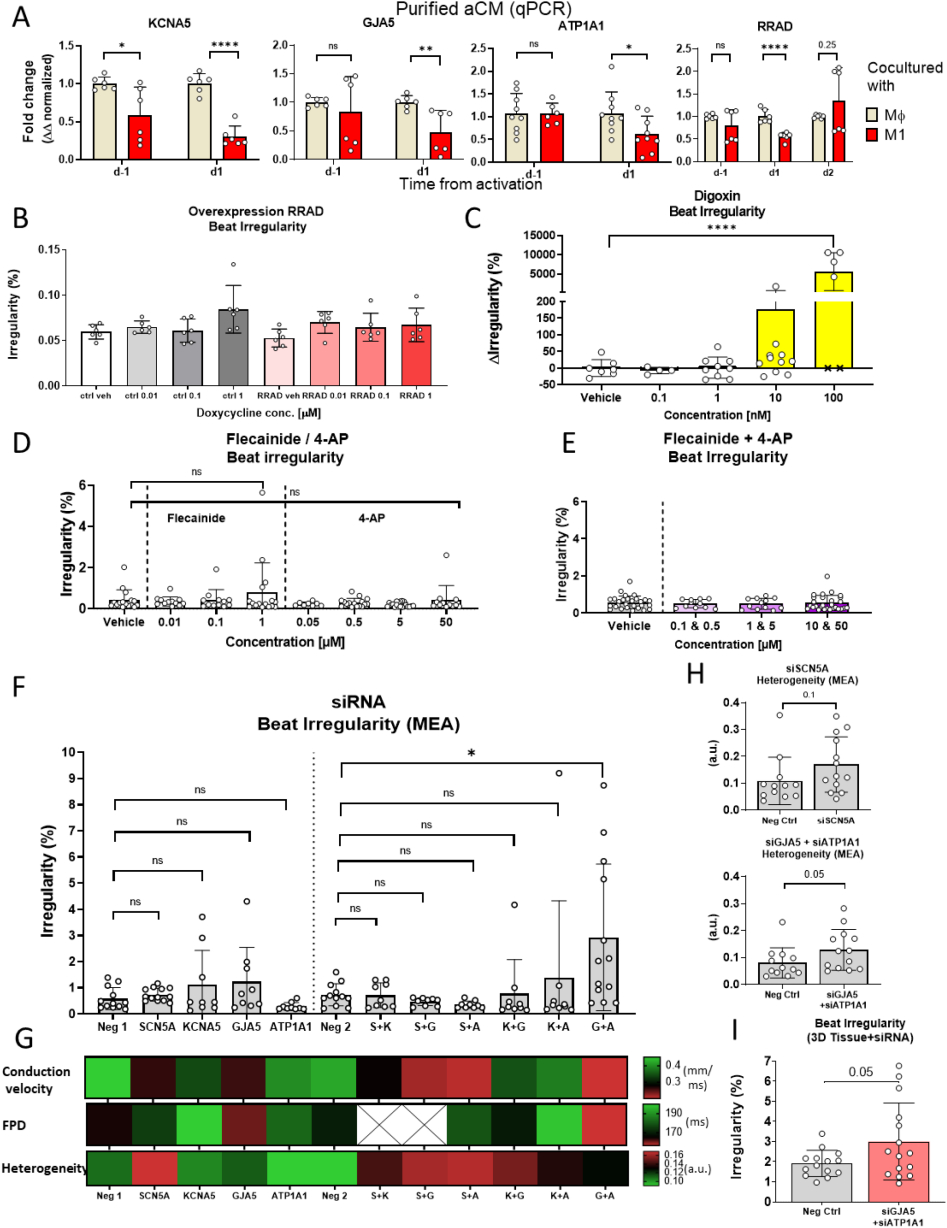
Combined inhibition of *GJA5* and *ATP1A1* results in pro-arrhythmogenic phenotype. **(A)** qPCR expression of *KCNA5*, *GJA5*, *ATP1A1* and *RRAD* in purified aCM after coculture with either M1 or Mϕ, before (d-1) and after (d1, d2) activation, normalized to GAPDH and Mϕ expression levels (KCNA5, GJA5, RRAD: n=6; ATP1A1: n=9,6,9,9/N=3; unpaired student t-test). MEA recordings of beat irregularity in aCM monolayers **(B)** transduced with doxycycline inducible RRAD or negative control lentiviral vectors (n=6/N=1), **(C)** Na+/K+ ATPase inhibitor digoxin ((x) denote cessation of beating and spike detection) (n=6,4,9,11,6/N=1, Mann-Whitney test), **(D)** I_Na_ inhibitor flecainide or I_Kur_ inhibitor 4-AP (n=16,12,12,12,8,16,20,12/N=3, Mann-Whitney test), **(E)** combination of both inhibitors flecainide and 4-AP (n=31,11,13,31/N=3), and **(F)** siRNA against *SCN5A* (S), *KCNA5***(K)**, *GJA5***(G)** and *ATP1A1***(A)** individually and in combination. Scrambled siRNA used as negative control, at matched concentrations to targeted siRNA denoted as single dose (Neg 1) and double dose (Neg 2) (N = 12,13,9,9,9,12,9,9,9,9,9,13/N=3, One Way ANOVA). **(G)** Heat map showing electrophysiological parameters from MEA recordings of siRNA treated aCM monolayers. **(H)** Bar graphs showing heterogeneity for siRNA treated conditions compared to negative control siRNA treated (Neg Ctrl) 2D aCM, showing an increase in conduction heterogeneity from *GJA5* and *ATP1A1* inhibition (n=12,13/N=3, Mann-Whitney test). **(I)** Beat irregularity of 3D aCM+cfb+Mφ tissues treated with siRNA for *GJA5* and *ATP1A1* or negative control siRNA (Neg Ctrl) showing increase irregularity for siRNA treated tissues (n=14,15/N=3, unpaired student t-test). ns, not significant, *P<0.05, **P<0.01, ****P<0.0001.

Previous RNA-seq analysis (8) identified that key electrophysiology-related genes were dysregulated in aCM and M1 monolayer cocultures and were restored through drug-mediated suppression of M1 activation. The genes encoding key electrophysiology proteins with restored expression (*SCN5A*, *KCNA5*, *GJA5*, *ATP1A1* and *RRAD*) were hypothesized to be involved with inflammation-induced beat irregularity. qPCR analysis confirmed dysregulation of some of these genes in the aCM and M1 coculture, identifying a time-dependent effect with reduction of genes 1 day after activation (ΔΔ fold change: 0.3, *SCN5A*, P<0.0001; 0.3, *KCNA5*, P<0.0001; 0.45, *GJA5*, P<0.01; 0.6, *ATP1A1*, P<0.05; **Figure 3A**, **Figure 5A**).

*RRAD* expression, known to be upregulated due to M1-inflammation in iPSC *in vitro* models (8) and in clinical AF (15), was upregulated at d2 after activation (ΔΔ fold change: 1.3, P = 0.25; **Figure 5A**). To investigate whether *RRAD* upregulation leads to arrhythmia, aCM monolayers were transduced with a doxycycline inducible *RRAD* overexpressing lentiviral vector. *RRAD* overexpression levels under increasing doxycycline concentrations (0.01-1 µM) were confirmed through qPCR (**Supplementary Figure S5A**). However, increased *RRAD* expression did not lead to increased beat irregularity, as determined by MEA analysis (**Figure 5B**). Instead, overexpressed *RRAD* significantly increased spike amplitude and conduction velocity (**Supplementary Figure S5B, C**), suggesting involvement in a reparative mechanism. This could explain the delayed expression increase observed in **Figure 5A** and enhanced expression in clinical datasets.

To investigate other potentially causative gene candidates for beat irregularity, mechanistic analysis was performed through selective inhibition of target ion channel genes [*SCN5A* (**Figure 3A**), *KCNA5* and *ATP1A1* (**Figure 5A**)]. Drug-induced inhibition was tested first. Treatment with the Na/K pump (transcribed by *ATP1A1)* inhibitor, digoxin, increased beat irregularity in aCM (8,388% ± 2,976 increase at 100 nM *vs*. vehicle) and led to cessation of contraction in individual wells at 100 nM concentration (**Figure 5C**). Digoxin further caused a dose-dependent increase in beat rate (10.6% ± 2.5 increase at 10 nM *vs*. vehicle, **Supplementary Figure S5D**). This could be due to a less negative membrane potential cause by Na/K pump inhibition (34), resulting in a shorter time to reach the depolarization potential threshold. *SCN5A* transcribes the ion channel Nav1.5, while *KCNA5* transcribes the potassium channel responsible for the I_kur_ current. Treatment with the Nav1.5 antagonist, flecainide, and I_kur_ blocker, 4-AP, did not increase beat irregularity in aCM, neither individually (**Figure 5D**) nor in combination (**Figure 5E**). Flecainide did reduce spike amplitude (-100% at 10µM, **Figure 3B**) and conduction velocity (-18% at 1µM, **Supplementary Figure S5E**), while 4-AP did not affect either (**Supplementary Figure S5E**). This could be due to flecainide acting on the sodium channel, inhibiting sodium influx (e.g., spike amplitude) and in turn reducing the excitability and therefore conduction of the aCM. Treatment of aCM+Mφ cocultures with flecainide and 4-AP also did not result in a significant increase in beat irregularity (**Supplementary Figure S5F**). Further, compound inhibition using flecainide and 4-AP did not affect *RRAD* expression levels, indicating RRAD transcription to not be directly modulated by reduction in sodium or potassium currents. This suggested that functional inhibition of the ion channels transcribed by ATP1A1, but not SCN5A or KCNA5, could lead to arrhythmia induction similar to the observed inflammation-induced phenotype.

To study whether downregulation of the target genes by genetic modification would recapitulate the observed increase in beat irregularity of the disease model, aCM monocultures were next treated with siRNAs. Successful siRNA-mediated knock-down of target genes was confirmed using qPCR following functional MEA analysis, showing significant mRNA level reduction for all target genes (knock-down 53.5% ± 15.1 *SCN5A*, 70% ± 16.9 *KCNA5*, 35.3% ± 19.5 *GJA5*, 85.5% ± 15.5 *ATP1A1*; **Supplementary Figure S5H**).

Knock-down of individual genes, as well as combinatorial knock-down of gene pairs were analyzed using MEA (**Figure 5F**). Single gene manipulation did not result in a significant increase in irregularity compared to negative control siRNA. Nevertheless, combined knock-down of *GJA5* and *ATP1A1* resulted in significant increase in beat irregularity compared to negative control siRNA (402.9% ± 371.3). MEA data from siRNA-treated aCM monocultures were analyzed for additional electrophysiological parameters, including conduction velocity, FPD and conduction heterogeneity (**Figure 5G**). Knock-down showed expected functional results, including *KCNA5* downregulation slowing potassium-dependent repolarization by 22%, as well as independent *SCN5A* and *GJA5* downregulation reducing conduction velocity (42.4% for *SCN5A* and 43.2% for *GJA5*) through reduced excitability and gap junction disturbance, respectively. Combination of *GJA5* and *ATP1A1* knock-down consistently promoted a pro-arrhythmic phenotype (**Figure 5F, G**). Besides causing beat irregularity, this knock-down combination also increased conduction heterogeneity (18%), reduced conduction velocity (52.4%) and shortened FPD (8%). Conduction heterogeneity was further analyzed and showed no significant increase for single gene siRNA inhibition, with *SCN5A* having the highest absolute increase (0.17 a.u. ± 0.1 (si*SCN5A*) *vs*. 0.11 a.u. ± 0.09 (Neg Ctrl); **Figure 5H**). Combined inhibition of *GJA5* and *ATP1A1* increased heterogeneity (0.13 a.u. ± 0.08 (si*GJA5+*si*ATP1A1*) *vs*. 0.08 a.u. ± 0.06 (Neg Ctrl). *GJA5* and *ATP1A1* downregulation were confirmed as pro-arrhythmic using the 3D model of aCM+cfb+Mφ, where siRNAs caused significant increase in beat irregularity compared to negative control (56.8% ± 36.3; **Figure 5I**).

Results from drug and genetic modification studies suggested a causative mechanism for initiation of beat irregularities in aCM through a combined downregulation of *GJA5* and *ATP1A1.* Disruption of these genes by M1, as seen in previous results (**Figure 5A**), could therefore be mechanistically linked to the beat irregularities caused by M1-induced inflammation.

### Down-regulation of Na+/K+ pump and conductivity increase beat irregularity in *in silico* hiPSC-CM and adult atrial CM tissue models

**Figure 6:**
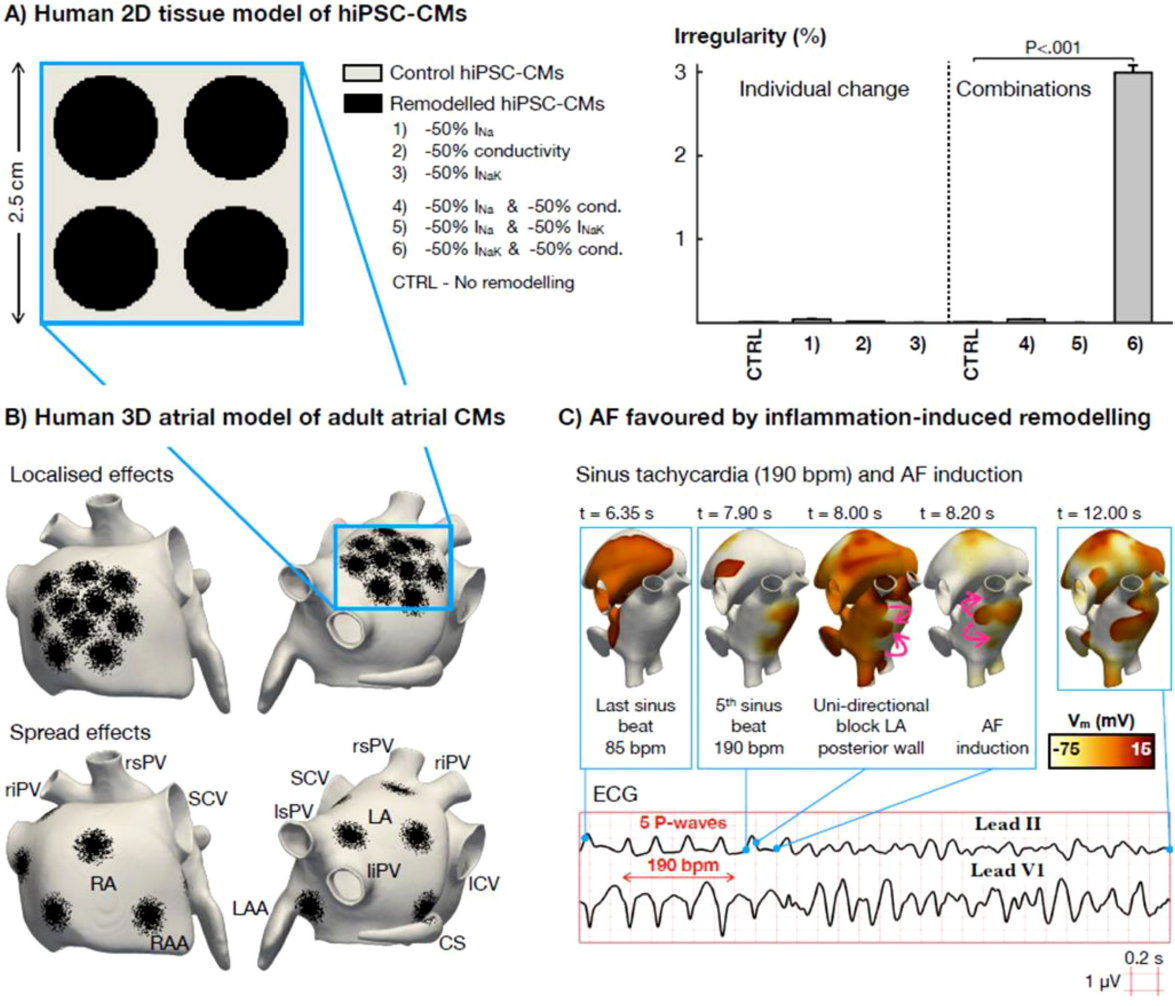
*In silico* investigation of the proarrhythmic potential of inflammation-induced electrophysiological remodeling. **(A)** Comparison of beating irregularity in an *in silico* hiPSC-CM tissue between control conditions (CTRL – no remodeling) and six scenarios of electrophysiological remodeling, comprised of 50% reduction of 3 separate parameters (I_Na_ sodium current, conductivity e.g., connexin connectivity and I_NaK_ Na+/K+ ATPase current) and their combinations (One Way ANOVA). **(B)** Schematic representation of a human 3D model of the atria composed of adult atrial cardiomyocytes (CMs). Several patches simulating electrophysiological remodeling were tested, either localized or spread across the atria, to reproduce the spatial heterogeneity of the inflammatory response. Abbreviations. RA-LA: right and left atrium; RAA-LAA: RA and LA appendage; SCV-ICV: superior and inferior cava vein; rs-ri-ls-li-PV: right superior, right inferior, left superior and left inferior pulmonary vein; CS: coronary sinus. **(C)** Representative case of atrial fibrillation (AF) induced by sinus tachycardia (i.e., 190 beats per minute) and favored by the heterogeneous repolarization substrate resulting from inflammation-induced electrophysiological remodeling. Pink arrows represent conduction wavefront reentry.

*In silico* computer modeling was used as an orthogonal method to confirm the *in vitro* results and apply them to a relevant human whole organ model. Initially, electrophysiological remodeling observed due to combined GJA5 and ATP1A1 knock-down (as shown in **Figure 5F**) was recapitulated *in silico* using hiPSC-CM tissue slabs (19). **Figure 6A** illustrates beat irregularity effects in the *in silico* hiPSC-CM tissue model after simulating individual downregulation of the fast Na^+^ current (I_Na_), Na+/K+ pump (I_NaK_), and tissue conductivity (representing collective connexin activity), as well as the combined effect of paired parameter down-regulation. In alignment with *in vitro* hiPSC-CM 3D tissue data, simulations revealed a significant increase in beat irregularity only after combined down-regulation of I_NaK_ and tissue connectivity by 50% (**Figure 6A**), while individual parameter down-regulations were not sufficient to induce perturbations. Simulation results for different geometries (0.25–1 cm radial patches covering 50% of tissue area) and intensities (25-50% knock-down) of inflammation-induced remodeling are presented in **Supplementary Figure S6**, with 0.5 cm patches and 50% knock-down demonstrating optimal effects. Interestingly, 50% knock-down of I_NaK_, and I_NaK_ in combination with reduced I_Na_ and conductivity, caused cessation of beating when smaller patches of 0.25-0.34 cm radius were used in simulations.

*In silico* modeling in hiPSC-aCM tissue validated the arrhythmia induction mechanism observed *in vitro*. Next, the proarrhythmic potential of paired GJA5 and ATP1A1 alteration was investigated in a human whole-organ model of the atria (20). Similar to the hiPSC-CM computer model, spatial heterogeneity of the inflammatory response was simulated in the human whole-atria *in silico* model. Electrophysiological remodeling was applied either as localized infiltration, primarily located in the venous portion of the right and left atrium (**Figure 6B**, top) or spread-out across the atria (**Figure 6B**, bottom).

In localized as well as spread infiltration, paired downregulation of I_NaK_ and tissue conductivity produced prolongation of atrial repolarization (**Supplementary Figure S7**). Prolongation of refractoriness occurred only in regions affected by the electrophysiological remodeling, creating a heterogeneous repolarization substrate. Thus, in the event of sinus tachycardia (i.e., 190 beats per minute), the heterogeneous repolarization substrate was sufficient to induce AF even in the absence of ectopic stimuli (**Figure 6C**). AF was elicited for both localized and spread infiltration of the electrophysiological remodeling, but not in control conditions (i.e., absence of remodeling). This demonstrates a causative effect towards AF initiation due to downregulation of I_NaK_, representing *ATP1A1* activity, and conductivity, representing *GJA5* activity. Alignment of results between *in silico* human adult whole-atria and hiPSC-CM tissue, as well as *in vitro* hiPSC-CM tissue demonstrate the validity of the *in vitro* model and collectively present a new potential mechanism for AF initiation.

## Discussion

**Figure 7:**
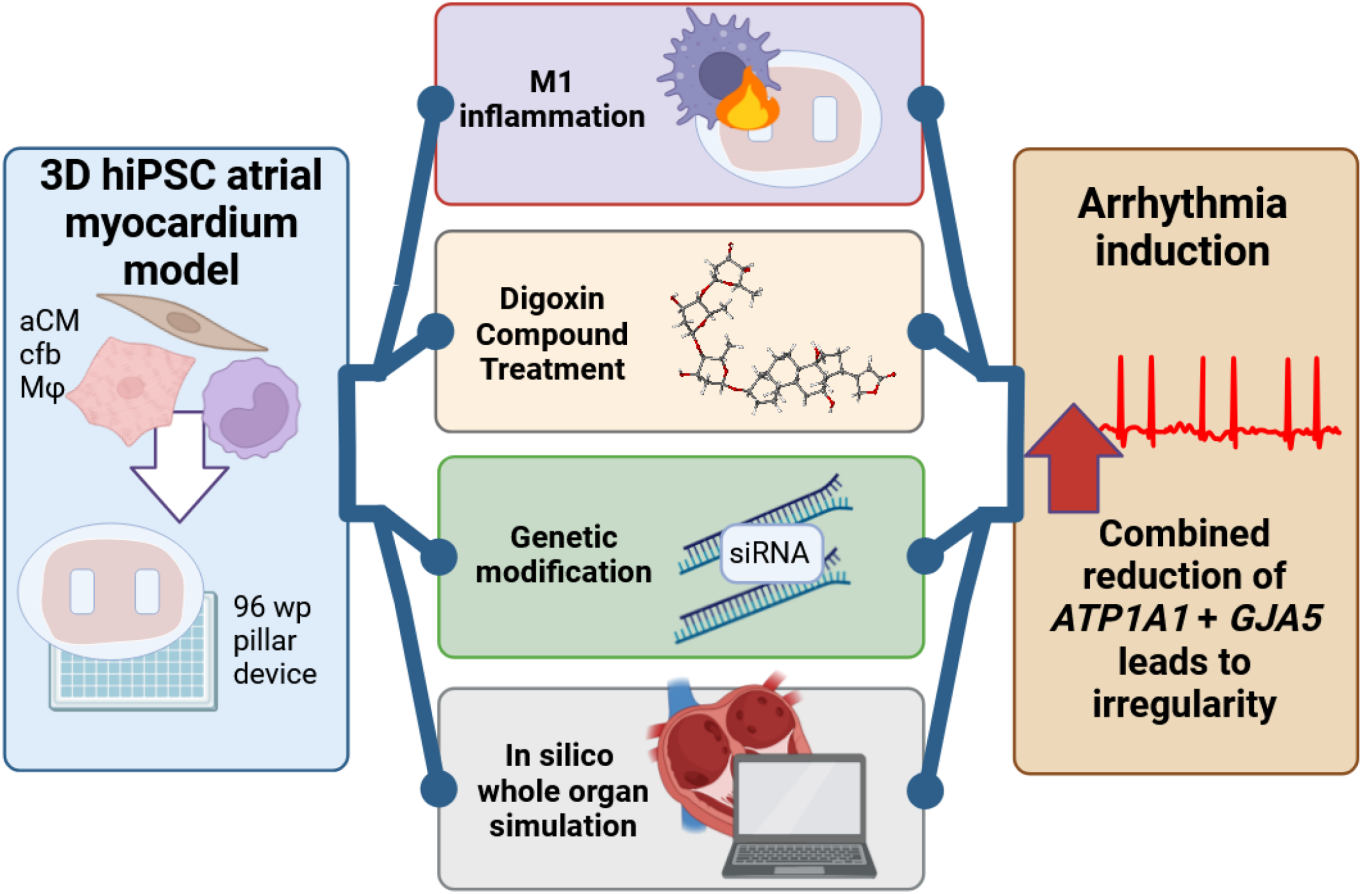
Schematic summary of findings.

Inflammation-caused cardiac dysfunction has been a long-observed phenomenon, like contraction dysfunction is known to be initiated by inflammatory processes, including sepsis (9). AF, as well, has been shown to cause reduced contraction and impaired systolic Ca^2+^ transients, observed in tachypaced AF dog (35) and goat models (29) and reduced contraction in human patient tissues (36, 37). Similarly, inflammation has been increasingly shown to plausibly cause arrhythmia, with macrophages proposed as disease related in murine models through a variety of proposed mechanisms such as IL-6 or IL-1β signaling. Click or tap here to enter text (7, 8, 30, 38, 39). This study presented reduced contraction and emergent arrhythmia as directly correlated to macrophage-mediated inflammation, identifying specific genes (*SCN5A*, *GJA5*, *ATP1A1*) as causative of the dysfunction, in 3D physiologically relevant aCM human tissue models constructed from 3 donor hiPSC lines. Having identified a shortlist of genes differentially expressed in aCM due to co-culture with activated M1 (8), the direct role of each gene in electrophysiological perturbances was investigated pharmacologically, via siRNA and overexpression genetic modification, and *in silico* (**Figure 7**). All validation methods indicated a direct involvement of *GJA5* and *ATP1A1* in pro-arrhythmia. This study thereby proposes a new, distinct mechanism of how potentially cardiac tissue resident macrophage activation can directly lead to AF even in the absence of ectopy.

*GJA5*+*ATP1A1* downregulation was identified as a potential mechanism of M1 inflammation-induced arrhythmia. The 3D tissue *in vitro* model demonstrated an increase in irregularity and reduction of contraction amplitude caused by M1 inflammation. These findings were validated through 3 alternative methodologies, all confirming the mechanism that involvement of inter-cellular connections (particularly created through *GJA5*-transcribed Cx40) and of sodium ion flux (particularly through the *ATP1A1*-transcribed Na/K pump) leads to arrhythmia induction. Whole organ *in silico* modeling confirmed these changes to lead to atrial fibrillation in the absence of ectopic activity.

Individually, each of the genes highlighted in this study have previously been associated with cardiac function. Namely, a heterozygous, deleterious variant of *SCN5A* was identified as fatal in a case of myocarditis (40), and downregulation of this gene has been recorded in the atria of heart failure patients (41). In this study, *KCNA5* downregulation increased FPD as expected (**Figure 5F**) but was not arrhythmogenic. This could be, for example, due to functional reduction in I_kur_ current being compensated by other, non-affected potassium currents (I_ks_, I_kr_) (42). *GJA5* (transcribing the atrial-specific, Connexin 40), is known to be downregulated by inflammation associated viral infections like COVID-19, showing a reduction in SARS-COV-2 infected hiPSC CMs (43). Further, rare variants in GJA5 have been associated with early-onset lone AF (44). *ATP1A1* (Na^+^/K^+^ ATPase), is shown to be highly downregulated by about 50% in the atria of heart failure patients (41). Finally, reduced function of *ATP1A1* through mutation has been shown to affect cardiac rhythm, by prolonging QT interval and causing bradycardia in zebrafish (45), and downregulation has been implicated in a Pgc-1α deficient mouse model of AF (46).

To the authors’ knowledge, combined down-regulation of these genes, and particularly in the context of AF, has not been investigated to date. In this study, the cellular phenotypes observed by inflammation-induced downregulation of *GJA5* and *ATP1A1* genes resulted in increased irregularity, which was reproduced *in vitro* by drug-induced and genetic methodologies, as well as *in silico* in hiPSC and adult atria tissue models.

Further, observed effects included increased conduction heterogeneity on a tissue level (**Figure 5H**), reduced maximum diastolic potential and increased AP instability on a cellular level (**Figure 4D, E**, **Supplementary Figure S4C**). The observed variation in AP (APD_90_ and AUC_90_), alternans and incomplete repolarizations, were possibly related to *GJA5* and *ATP1A1* dysregulation and related heterogeneity and automaticity. The need for combined downregulation of both genes for arrhythmia to emerge is notable and might point to individual aCM electrophysiological instability having to be exacerbated through lack of conductivity with neighboring cells for arrhythmia to occur, similarly to how in AF a trigger requires a substrate for the disease to emerge (47). The identified influence of heterogeneity in *in silico* modeling might explain the observed variance in beat irregularity recorded in the *in vitro* disease models. This in turn suggests that heterogeneity through tissue composition could influence the initiation of beat irregularity. Tissue heterogeneity has long been understood to play a key role in AF perpetuation (48). Nevertheless, this study utilized tissues with the same cellular composition (aCM+cfb+Mφ) as negative controls to the disease model, isolating inflammation-induced cellular changes as the instigator for beat irregularity and other electrophysiological abnormalities.

The calcium channel regulator, *RRAD*, has previously been linked to arrhythmia through gain of function mutation in an hiPSC model of Brugada syndrome (49), and was moreover reported as significantly upregulated in patient tissue samples of paroxysmal and persistent AF in a clinical study (CATCH ME) (15). This study therefore investigated RRAD overexpression as possibly pro-arrhythmic. However, findings indicated that wild-type *RRAD* overexpression might be proposed as a reparative mechanism, based on improved spike amplitude and conduction velocity (**Supplementary Figure S5B, C**). A recent study identified a missense mutation of *RRAD* to reduce sodium current amplitude, suggesting a direct connection between the two through a yet unidentified mechanism (50).

The proposed beat irregularity mechanism highlights additional potential therapeutic targets against inflammation-related arrhythmia and cardiac dysfunction. The restoration of cellular phenotypes through immunosuppressants (hydrocortisone) further supports this possibility. Such medication could be combined or substituted by gene therapies for ATP1A1 and/or GJA5.

It has to be stated that the mechanism shown as causative in this study does not preclude other potential mechanisms. While the specific inhibition of target genes resulted in occurrence of disease-associated phenotypes, the transcriptional effect of inflammation and macrophage coculture on aCM is extensive (8). Therefore, further studies on the interplay between aCM and inflammation function is necessary.

The hiPSC model is limited through the immature physiology of the hiPSC-derived cells, which includes a presence of fetal isotype of *SCN5A* and reduced upstroke velocity (51) when compared to human adult CM. However, the significant increase in upstroke in the 3D model *vs*. 2D aggregates shows increased maturation and, therefore, physiological relevance. Regarding the use of a tissue model, it has to be stated that although the small size of tissues allows for high throughput experimentation, its size decreases the likelihood of some AF phenomena occurrence (notably, reentries or rotors). Furthermore, both control and test tissues might not be homogenous in terms of cell type distribution. The presented *in silico* model of hiPSC-CM demonstrates the impact of heterogeneity, therefore tissue morphology of *in vitro* models could benefit from further refinement. As part of the 3D tissue models, the utilized cfb presented limitations as well, particularly in their characterization. Although a cardiac mesoderm-dependent differentiation protocol was implemented through Wnt signaling modulation, more detailed characterization with markers more specific to cardiac fibroblasts and activated myofibroblasts (e.g., DDR2 or Periostin, respectively), could enhance cell type resolution and validation of cardiac fibroblast presence in the modelClick or tap here to enter text (52, 53). Nevertheless, the cardiac mesoderm-based protocol used here resulted in cells with a stereotypical spindle-like phenotype, transcription (COL1A1, MMP2), and protein expression (collagen, vimentin) suggesting a fibroblast identity (**Supplementary Figure S1E–G, S2B**). For this study, the primary cfb function was structural integrity and ECM deposition to allow successful tissue formation. Beyond this, the inclusion of fibroblasts within cardiac EHTs is known to have physiological relevance by increasing tissue cohesion, supporting contraction force generation and more closely mimicking the human tissue makeup (54). Further, fibroblasts have been shown to affect cardiac electrophysiology and maturation (55), and form gap junctions (Connexin 43) and interact via paracrine factors with cardiomyocytes (56). Cardiac fibroblasts and their interplay in inflammation is further suggested to be relevant for pathophysiological process, including electrophysiology (57). While the latter were not the focus of this study, they could form the subject of future investigations.

Similarly, further subtype characterization could be performed for cardiac M1 macrophages to validate their identity, via lineage and activation markers such as MHC-II, CD68 or CD163 (58). In this study, the retained expression of CX3CR1 in tissues (**Figure 1E**), as well as prior characterization of our differentiation method showing CD86 expression and IL-6 secretion in derived cells (8), suggests a resident M1 macrophage type. Finally, as the presented models were *in vitro* and *in silico*, they lacked certain complexities of *in vivo* models, including a whole organ system, innervation and other immune cell types.

Nevertheless, the presented *in vitro* model of a 3D, atrial tissue allows for further research into additional aspects of AF, as well as related comorbidities, including arrhythmia-causing infection (e.g. COVID-19 (59)), fibrosis and disease progression. The presented 3D model showed the benefits of using injection molding as a production technique to fabricate pillar devices, which sustained myocardium tissue formation and analysis. Injection molding enabled the reliable production of pillar devices which were instrumental in analyzing tissue contractions. The described method was a reliable and reproducible way for tissue formation, manipulation and readout.

In conclusion, the study presents a novel 3D atrial inflammation model, which recapitulated macrophage-initiated, functional pathophysiologies such as reduced contraction, action potential alternans and beat irregularities. Transcriptional changes in aCM were identified to be caused by pro-inflammatory macrophage activation, which resulted in electrophysiological remodeling of aCM. *SCN5A* was found to be downregulated, correlating to reduced upstroke velocity and ultimately reduced contraction amplitudes. Further, inflammation-induced, combined reduction of *GJA5* and *ATP1A1* resulted in increased conduction heterogeneity, beat irregularity and a pro-arrhythmic phenotype. This was corroborated by drug treatment, genetic modification and, importantly, human-based computer modelling and simulation, finding the combined downregulation in connection with heterogeneous distribution to enhance inducibility of simulated AF. These findings highlight a new mechanism for inflammation-mediated AF induction and provide new therapeutic avenues for this prevalent disease.

## Data Availability

The raw data supporting the conclusions of this article will be made available by the authors, without undue reservation.

## Supplementary material

Supplementary Figure 1 hiPSC-differentiation yielded atrial-like cardiomyocytes (aCM), Mϕ macrophages, and cardiac fibroblast-like cells expressing subtype specific biomarkers. **(A)** Flow cytometry plots of hiPSC-derived Mϕ macrophages from three hiPSC lines at harvest (d0), showing expression of CD45, CD11b and CD14, leukocyte and monocyte/macrophage markers (60). (**B)** Flow cytometry images of MLC2a and cTnT double staining for aCM (NC-196) at d14 and d28 post differentiation, showing subtype-specific expression of MLC2a (61) retained over time. (**C)** RT-qPCR expression levels for atrial subtype marker (62) *NR2F2* for aCM and vCM at 14 days post differentiation, values ΔΔ Ct normalized to GAPDH and vCM expression levels (n=3/N=1, unpaired student t-test). (**D)** Flow cytometry plots showing double staining for atrial-specific marker COUP-TF II (62) and for cardiac marker cTnT in aCMs, on day 14 of differentiation. (**E)** Brightfield images of hiPSC-derived cfb. (Scale bar: 200 µm) (**F) G**ene expression in cfb and hiPSC for fibroblast genes (63) *COL1A1* and *MMP2*, normalized to GAPDH and hiPSC expression (n=3/N=1, unpaired student t-test). (**G)** IF image of cfb stained for mesenchymal cell marker Vimentin (24) and endothelial cell marker CD31 (24) showing absence of endothelial biomarker CD31. (Scale bar: 200 µm) (**H)** IF images of cfb and smc stained for Vimentin and smooth muscle cell and fibroblast activation marker a-Actin (24, 64) absence of smc-like a-Actin expression in cfb. (Scale bar: 200 µm). ***P<0.001; ****P<0.0001 Abbreviations: human induced pluripotent stem cells (hiPSC), atrial cardiomyocytes (aCM), ventricular cardiomyocytes (vCM)

Supplementary Figure 2 M1 activation results in reduced collagen deposition in 3D tissues. **(A)** Sharp electrode recordings of single cell aCMs in 3D tissues consisting of aCM+cfb (n=21/N=4) compared to 2D monolayer of aCM (N = 32/N=3) for action potential amplitude and maximum diastolic potential (unpaired student t-test). **(B)** IF staining for ECM component, collagen 1, in an aCM+cfb+M1 tissue. **(C)** Collagen deposition at d1 after activation, measured by calculating the integrated fluorescence intensity divided by area, and normalizing to aCM+cfb (aCM only) (n=6,6,7,7/N=3), (One Way ANOVA). **(D)** Collagen deposition measured at d1 to d15 after M1 activation, calculated as integrated fluorescence intensity divided by tissue area (Mφ n=9, M1 n=11, N = 3; nested t-test), and indicating recovery of collagen deposition levels as the effects of M1 activation diminish over time. ns, not significant, *P<0.05, **P<0.01

Supplementary Figure 3 M1 inflammation affects contraction amplitude, sodium spike amplitude and action potentials of aCM in 3D tissues, while not affecting calcium decay slopes. **(A)** Brightfield image of aCM+cfb+M1 tissue at d1 after activation (NC-030) (Scale bar: 1000 µm). **(B)** Absolute contraction amplitudes (mean) of 3D tissues at d1 after activation, paired by experiment (n=50,56/N=6, paired student t-test). **(C)** Calcium transient analysis in 3D tissues, at d1 after activation, showing area under curve and falling slope times from 10 to 90% and 30 to 70% amplitude (n=18,20/N=3 unpaired student t-test). **(D)** Representative images of action potentials from sharp electrode recordings of individual aCM within tissues (NC-196), at d1 after activation. **(E)** Bar graphs of electrogram amplitude from MEA recordings in NC-196 2D cocultures, on d1 after activation. (n=36,38,45/N=3, One Way ANOVA). ns, not significant, *P<0.05, **P<0.01

Supplementary Figure 4 M1 inflammation resulted in increased beat irregularity, APD variance, less negative maximum diastolic potential and worsened conduction. **(A)** Brightfield image of aCM+cfb+M1 tissue at d1 after activation, in a sharp electrode recording flow chamber (Scale bar: 1000 µm). **(B)** Bar graphs of conduction velocity and beat irregularity in 2D NC-196 aCM coculture with M1 (aCM+M1), aCM coculture with Mφ (aCM+Mϕ) and aCM monoculture with M1 activation factors (aCM+act), from MEA recordings on d1 after activation (n=36,36,39/N=3 One Way ANOVA for conduction velocity, Kruskal-Wallis test for beat irregularity). **(C)** Sharp electrode recordings of maximum diastolic potential in individual aCM within tissues, at d1 after activation (n=34,24,30/N=4 One Way ANOVA). **(D)** APD_90_ variance from sharp electrode recordings of individual aCM within tissues, at d1 after activation (n=33,19,29/N=4 Kruskal-Wallis test). ns, not significant, *P<0.05, **P<0.01, ***P<0.001

Supplementary Figure 5 Compound treatment identifies inhibition of Na+/K+ pump activity in aCM to be pro-arrhythmic, while upregulation of *RRAD* or inhibition of Nav1.5 or I_kur_ to not affect beat regularity. **(A)** qPCR mRNA expression analysis for *RRAD*, in 2D aCM transduced with a doxycycline inducible RRAD or negative control lentiviral vector, normalized to GAPDH and ACTN housekeeping gene expression levels (n=3/N=1, One Way ANOVA intragroup, Two Way ANOVA intergroup). **(B)** MEA recordings of spike amplitude of 2D aCM transfected with a doxycycline inducible RRAD or negative control vector (n=6/N=1, paired student t-test). **(C)** MEA recordings of conduction velocity in aCM monolayers transduced with a doxycycline inducible RRAD or negative control lentiviral vector (n=6/N=1, paired student t-test). MEA recordings in 2D aCM cultures treated with z**(D)** digoxin, showing dose dependent increase in beat rate (n=13,4,4,5/N=1, One Way ANOVA), and **(E)** spike amplitude (n=4,3,5,4,3/N=1, One Way ANOVA) and **(F)** conduction velocity values of flecainide or 4-AP treated aCM (n=4,3,3,2,3,2,3,5,3/N=1, One Way ANOVA). **(G)** MEA recordings of beat irregularity in 2D aCM+M1, and aCM+Mϕ coculture treated with combination of flecainide and 4-AP, showing no significant increase in beat irregularity in treated cultures (n=56,53,41/N=3, Kruskal-Wallis test). **(H)** qPCR expression of *RRAD* in 2D aCM transduced with a doxycycline inducible RRAD or negative control vector and treated with flecainide and 4-AP, normalized to GAPDH and ACTN expression levels (n=3/N=1, Two Way ANOVA intergroup, unpaired student t-test 0 µM comparison). **(I)** qPCR expression in 2D aCM treated with siRNA against *SCN5A*, *KCNA5*, *GJA5*, *ATP1A1* or negative control (-), normalized to GAPDH and (-) expression levels (n=3,6,3,6,3,3,3,4/N=3, unpaired student t-test). ns, not significant, *P<0.05, **P<0.01, ***P<0.001, ****P<0.0001

Supplementary Figure 6 Degree of spatial remodeling complexity affects inducibility of beat irregularity by downregulation of electrophysiological parameters in an hiPSC-CM *in silico* model. Comparison of beating irregularity in an *in silico* hiPSC-CM tissue between control conditions (CTRL – no remodeling) and six scenarios of electrophysiological remodeling applied in four spatially heterogeneous configurations. Electrophysiological remodeling was applied to cover 50% of the whole tissue in circular patches of 1 cm radius (one circular patch), 0.5 cm radius (four circular patches), 0.34 cm radius (nine circular patches) and 0.25 cm radius (sixteen circular patches). For nine and sixteen circular patches, 50% I_NaK_ down-regulation prevented spontaneous beating of hiPSC-CMs. The blue box highlights the scenario included in **Figure 6**. Y-axis ranges automatically generated by simulation algorithms and vary between conditions tested.

Supplementary Figure 7 Heterogeneous repolarization substrate resulting from electrophysiological remodeling involved in inflammation. Consecutive snapshots of the transmembrane voltage (V_m_) in two representative 3D atrial models, one with localized and one with spread patches of remodeling. Remodeled patches prolonged the action potential duration locally, creating heterogeneous repolarization. Abbreviations. LA: left atrium; LAA: LA appendage; SCV-ICV: superior and inferior cava vein; rs-ri-ls-li-PV: right superior, right inferior, left superior and left inferior pulmonary vein; CS: coronary sinus.

Supplementary Table 1 List of Antibodies (ordered by mention).

Supplementary Table 2 qPCR Primers and corresponding sequences (ordered alphabetically).
