# Supplementary material for: GJA5 and ATP1A1 perturbations recapitulate inflammation-related beat irregularities in iPSC-based atrial myocardium tissue model
Source: Front Immunol. 2026 Mar 6;16:1719392. doi: 10.3389/fimmu.2025.1719392 (PMC13002374; doi:10.3389/fimmu.2025.1719392)
Supplement: Supplementary Figure 1 — hiPSC-differentiation yielded atrial-like cardiomyocytes (aCM), Mϕ macrophages, and cardiac fibroblast-like cells expressing subtype specific biomarkers. (A) Flow cytometry plots of hiPSC-derived Mϕ macrophages from three hiPSC lines at harvest (d0), showing expression of CD45, CD11b and CD14, leukocyte and monocyte/macrophage markers (60). (B) Flow cytometry images of MLC2a and cTnT double staining for aCM (NC-196) at d14 and d28 post differentiation, showing subtype-specific expression of MLC2a (61) retained over time. (C) RT-qPCR expression levels for atrial subtype marker (62) NR2F2 for aCM and vCM at 14 days post differentiation, values ΔΔ Ct normalized to GAPDH and vCM expression levels (n=3/N=1, unpaired student t-test). (D) Flow cytometry plots showing double staining for atrial-specific marker COUP-TF II (62) and for cardiac marker cTnT in aCMs, on day 14 of differentiation. (E) Brightfield images of hiPSC-derived cfb. (Scale bar: 200 µm) (F) Gene expression in cfb and hiPSC for fibroblast genes (63) COL1A1 and MMP2, normalized to GAPDH and hiPSC expression (n=3/N=1, unpaired student t-test). (G) IF image of cfb stained for mesenchymal cell marker Vimentin (24) and endothelial cell marker CD31 (24) showing absence of endothelial biomarker CD31. (Scale bar: 200 µm) (H) IF images of cfb and smc stained for Vimentin and smooth muscle cell and fibroblast activation marker a-Actin (24, 64) absence of smc-like a-Actin expression in cfb. (Scale bar: 200 µm). ***P<0.001; ****P<0.0001 Abbreviations: human induced pluripotent stem cells (hiPSC), atrial cardiomyocytes (aCM), ventricular cardiomyocytes (vCM) [file SupplementaryFile1.pdf]

GJA5 and ATP1A1 perturbations recapitulate inflammation-related beat irregularities in iPSC-based atrial myocardium tissue model

Thomas Hutschalik[1,2], Albert Dasí[3], Leto L Riebel[3], Maury Wiendels[4], Frederikus Bakker[5], Lucas J.A.M. Beckers[5], Koen C. Kriege[5], Susanne M. Valster[5], Roland C.M. Vulders[5], Ozan Özgül[2], Rémi Peyronnet[6], Blanca Rodriguez[3], Mariana Argenziano[1], Ulrich Schotten[2,7] and Elena Matsa[1,8,9,10]

[1]Ncardia Services B.V., J.H. Oortweg 21, 2333 CH Leiden, The Netherlands

[2]Dept. of Physiology, Cardiovascular Research Institute Maastricht, Maastricht, The Netherlands

[3]Department of Computer Science, University of Oxford, Oxford, United Kingdom

[4]Anatomy and Embryology, LUMC, Leiden, The Netherlands

[5]Department of Digital Standardization and Licensing Research, Intellectual Property and Standards, Royal Philips. High Tech Campus 4, Eindhoven, 5656 AE, the Netherlands

[6]Institute for Experimental Cardiovascular Medicine, University Heart Center Freiburg Bad Krozingen, and Faculty of Medicine, University of Freiburg, 79110 Freiburg, Germany

[7]Dept. of Cardiology, Maastricht University Medical Center, Maastricht, The Netherlands

[8]Cellistic, Rue Edouard Belin 2, 1435 Mont-Saint-Guibert, Belgium

[9]School of Biochemistry and Cell Biology, University College Cork, Cork, Ireland

[10]National Institute for Bioprocessing Research and Training, Dublin, Ireland

Corresponding author: Elena Matsa, ematsa@ucc.ie, School of Biochemistry and Cell Biology, University College Cork, Cork, Ireland

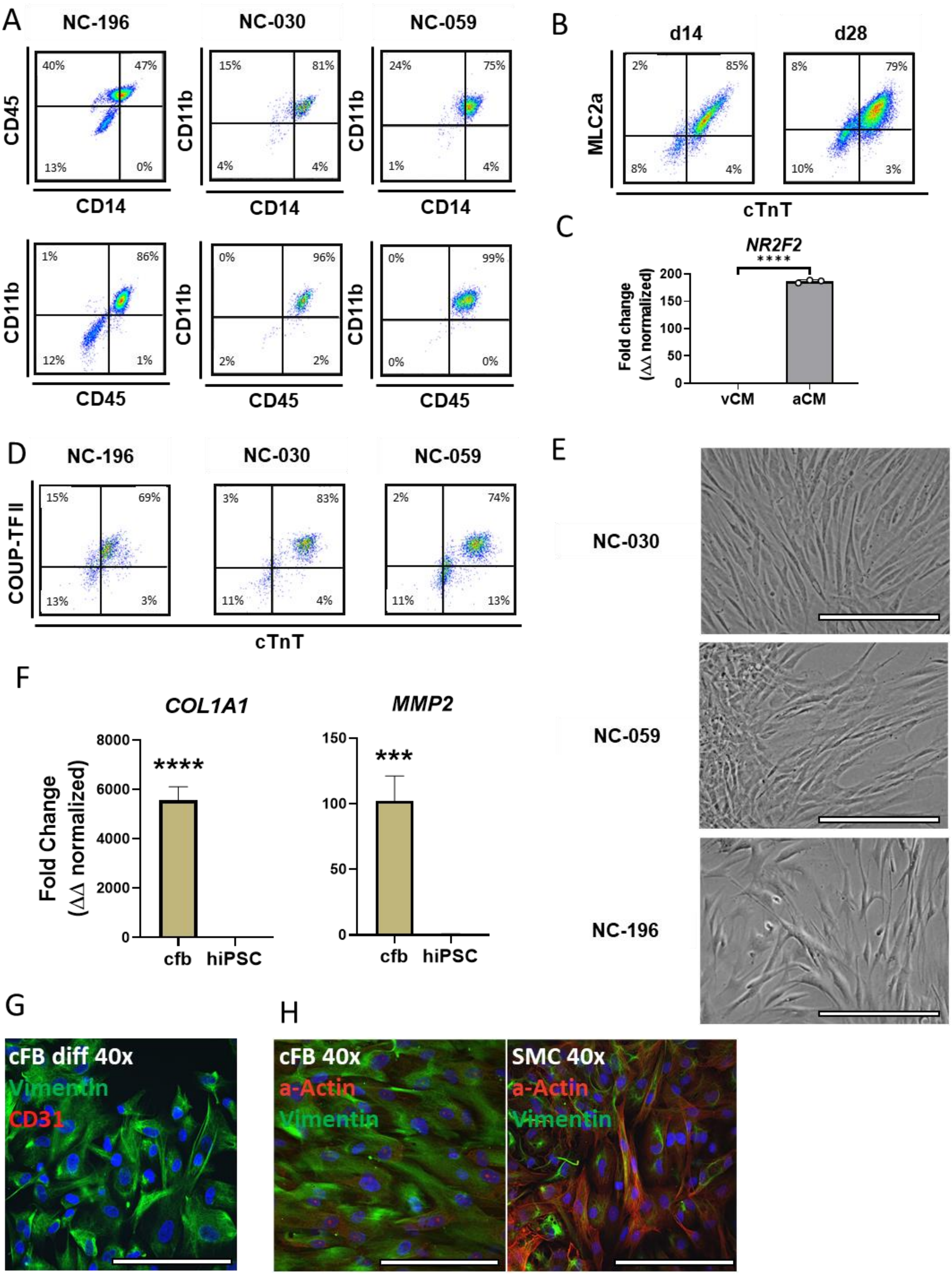
A
NC-196
NC-030
NC-059
CD45
CD14
CD11b
40%
47%
13%
0%
15%
81%
4%
4%
24%
75%
1%
4%
1%
86%
12%
1%
0%
96%
2%
2%
0%
99%
0%
0%
B
d14
d28
MLC2a
cTnT
2%
85%
8%
4%
8%
79%
10%
3%
C
NR2F2
****
Fold change (ΔΔ normalized)
200
150
100
50
0
vCM
aCM
D
COUP-TF II
15%
69%
13%
3%
3%
83%
11%
4%
2%
74%
11%
13%
E
NC-030
NC-059
NC-196
F
COL1A1
MMP2
Fold Change (ΔΔ normalized)
8000
6000
4000
2000
0
****
***
150
100
50
0
cfb
hiPSC
G
cFB diff 40x
Vimentin
CD31
H
cFB 40x
a-Actin
Vimentin
SMC 40x
a-Actin
Vimentin

Figure S1: hiPSC-differentiation yielded atrial-like cardiomyocytes (aCM), Mϕ macrophages, and cardiac fibroblast-like cells expressing subtype specific biomarkers

A) Flow cytometry plots of hiPSC-derived Mϕ macrophages from three hiPSC lines at harvest (d0), showing expression of CD45, CD11b and CD14, leukocyte and monocyte/macrophage markers[17]. **B)** Flow cytometry images of MLC2a and cTnT double staining for aCM (NC-196) at d14 and d28 post differentiation, showing subtype-specific expression of MLC2a[18] retained over time. **C)** RT-qPCR expression levels for atrial subtype marker[19] *NR2F2* for aCM and vCM at 14 days post differentiation, values ΔΔ Ct normalized to GAPDH and vCM expression levels (n=3/N=1, unpaired student t-test). **D)** Flow cytometry plots showing double staining for atrial-specific marker COUP-TF **I**I[19] and for cardiac marker cTnT in aCMs, on day 14 of differentiation. **E)** Brightfield images of hiPSC-derived cfb. (Scale bar: 200 µm) **F) G**ene expression in cfb and hiPSC for fibroblast genes[21] *COL1A1* and *MMP2*, normalized to GAPDH and hiPSC expression (n=3/N=1, unpaired student t-test). **G)** **I**F image of cfb stained for mesenchymal cell marker Vimentin[16] and endothelial cell marker CD31[16] showing absence of endothelial biomarker CD31. (Scale bar: 200 µm) **H)** **I**F images of cfb and smc stained for Vimentin and smooth muscle cell and fibroblast activation marker a-Actin[16,20] absence of smc-like a-Actin expression in cfb. (Scale bar: 200 µm). ***: P<0.001; ****: P<0.0001

Abbreviations: human induced pluripotent stem cells (hiPSC), atrial cardiomyocytes (aCM), ventricular cardiomyocytes (vCM)

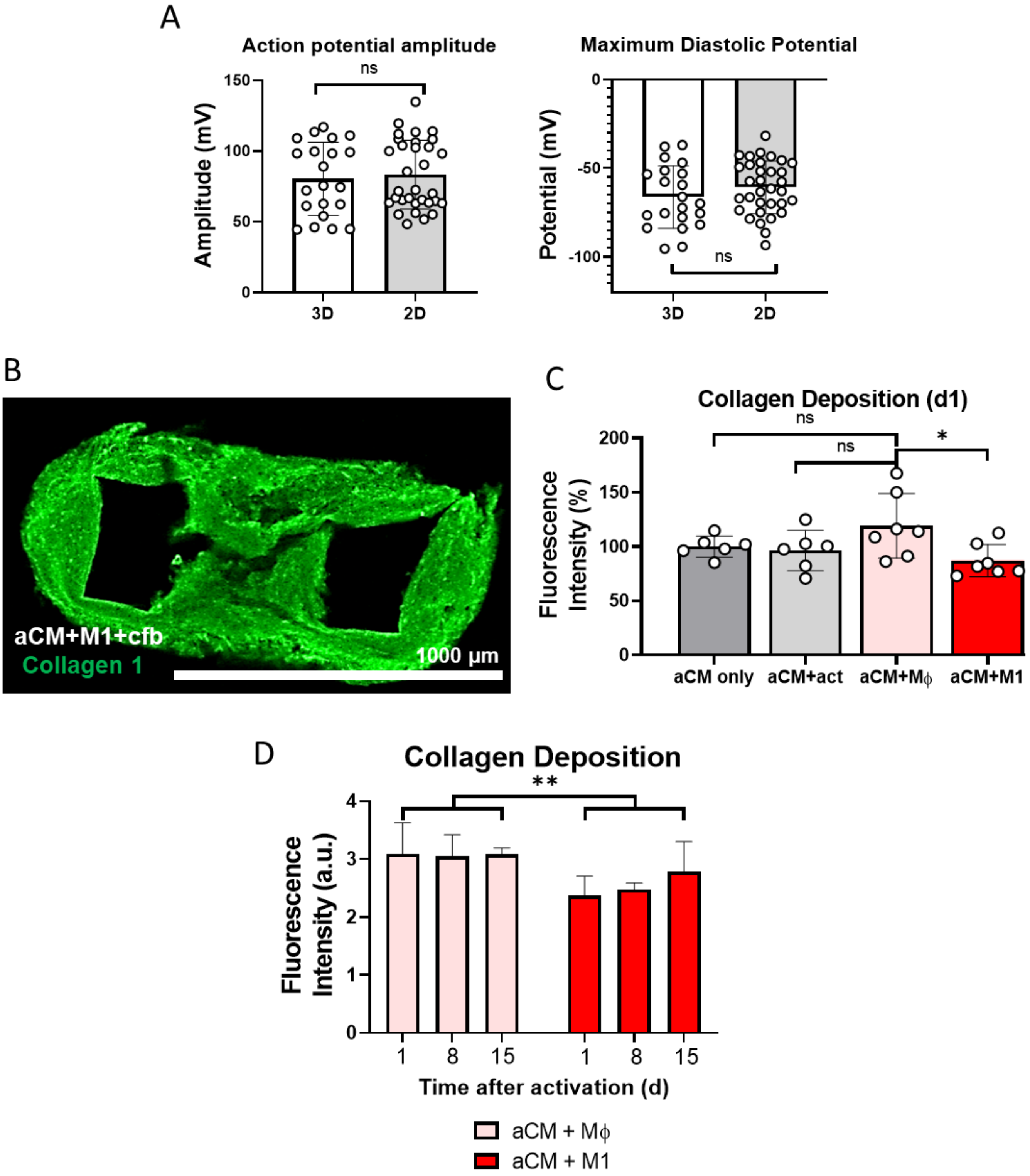

Figure S2: M1 activation results in reduced collagen deposition in 3D tissues

A) Sharp electrode recordings of single cell aCMs in 3D tissues consisting of aCM+cfb (n=21/N=4) compared to 2D monolayer of aCM (N=32/N=3) for action potential amplitude and maximum diastolic

potential (unpaired student t-test). **B)** IF staining for ECM component, collagen 1, in an aCM+cfb+M1 tissue. **C)** Collagen deposition at d1 after activation, measured by calculating the integrated fluorescence intensity divided by area, and normalizing to aCM+cfb (aCM only) (n=6,6,7,7/N=3), (One Way ANOVA). **D)** Collagen deposition measured at d1 to d15 after M1 activation, calculated as integrated fluorescence intensity divided by tissue area (Mφ n=9, M1 n=11, N=3; nested t-test), and indicating recovery of collagen deposition levels as the effects of M1 activation diminish over time. ns: not significant, *: P<0.05, **: P<0.01

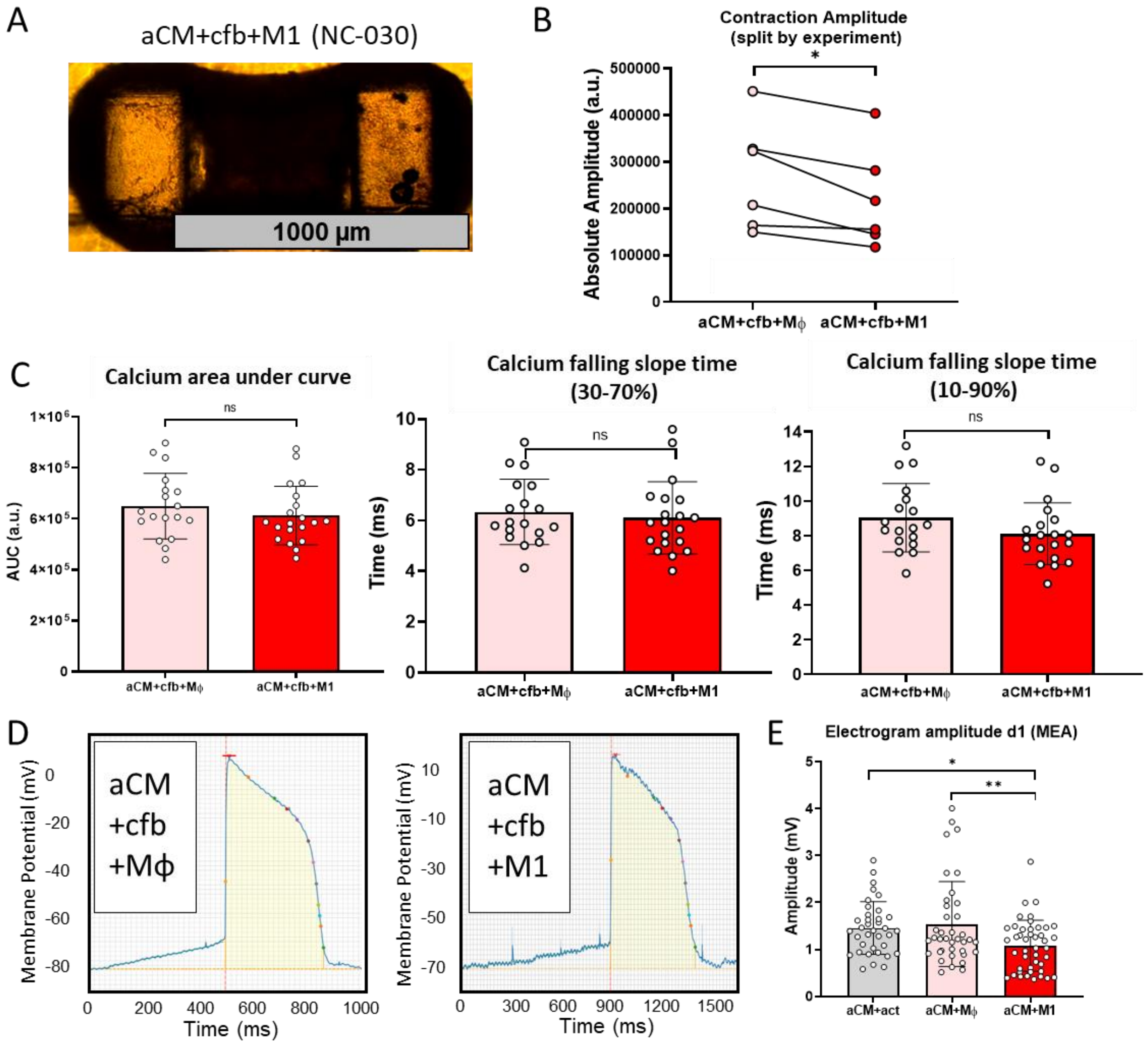

Figure S3: M1 inflammation affects contraction amplitude, sodium spike amplitude and action potentials of aCM in 3D tissues, while not affecting calcium decay slopes

A) Brightfield image of aCM+cfb+M1 tissue at d1 after activation (NC-030) (Scale bar: 1000 µm). **B)** Absolute contraction amplitudes (mean) of 3D tissues at d1 after activation, paired by experiment (n=50,56/N=6, paired student t-test). **C)** Calcium transient analysis in 3D tissues, at d1 after activation, showing area under curve and falling slope times from 10 to 90% and 30 to 70% amplitude (n=18,20/N=3 unpaired student t-test). **D)** Representative images of action potentials from sharp electrode recordings of individual aCM within tissues (NC-196), at d1 after activation. **E)** Bar graphs of electrogram amplitude from MEA recordings in NC-196 2D cocultures, on d1 after activation. (n=36,38,45/N=3, One Way ANOVA). ns: not significant, *: P<0.05, **: P<0.01

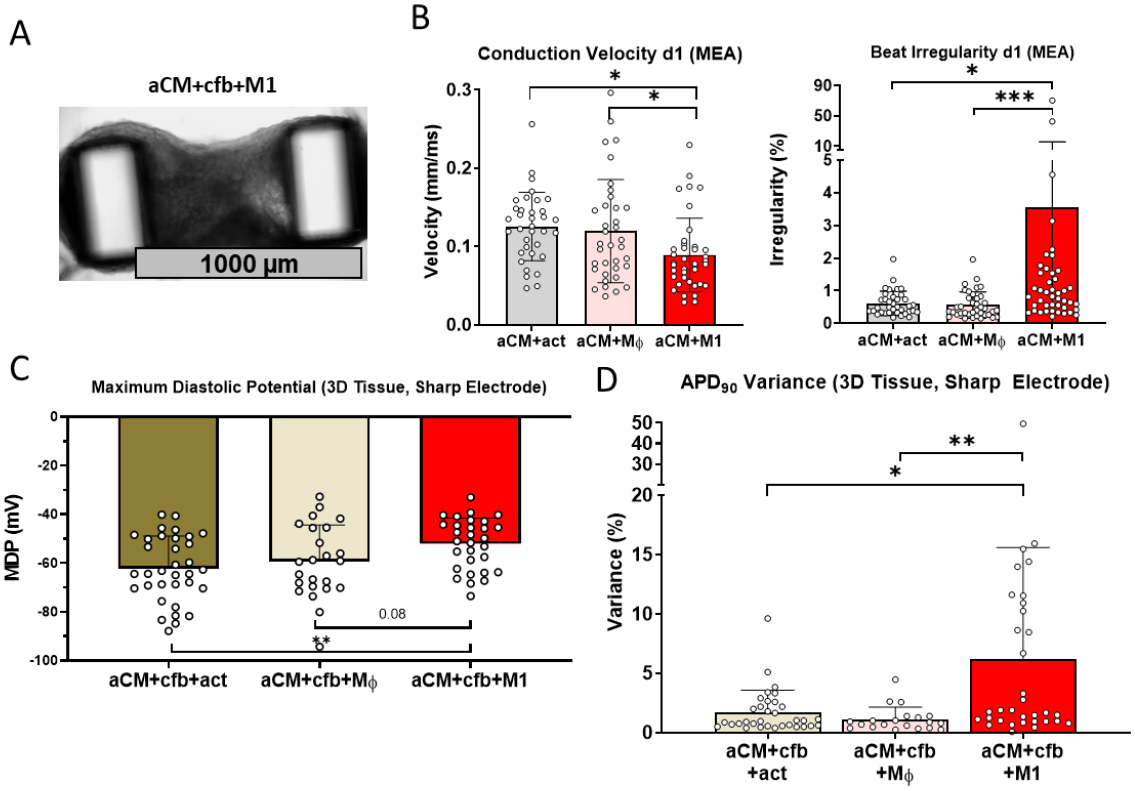

Figure S4: M1 inflammation resulted in increased beat irregularity, APD variance, less negative maximum diastolic potential and worsened conduction

A) Brightfield image of aCM+cfb+M1 tissue at d1 after activation, in a sharp electrode recording flow chamber (Scale bar: 1000 µm). **B)** Bar graphs of conduction velocity and beat irregularity in 2D NC-196 aCM coculture with M1 (aCM+M1), aCM coculture with Mφ (aCM+Mϕ) and aCM monoculture with M1 activation factors (aCM+act), from MEA recordings on d1 after activation (n=36,36,39/N=3 One Way ANOVA for conduction velocity, Kruskal-Wallis test for beat irregularity). **C)** Sharp electrode recordings

of maximum diastolic potential in individual aCM within tissues, at d1 after activation (n=34,24,30/N=4 One Way ANOVA). **D)** APD_{90} variance from sharp electrode recordings of individual aCM within tissues, at d1 after activation (n=33,19,29/N=4 Kruskal-Wallis test). ns: not significant, *: P<0.05, **: P<0.01, ***: P<0.001

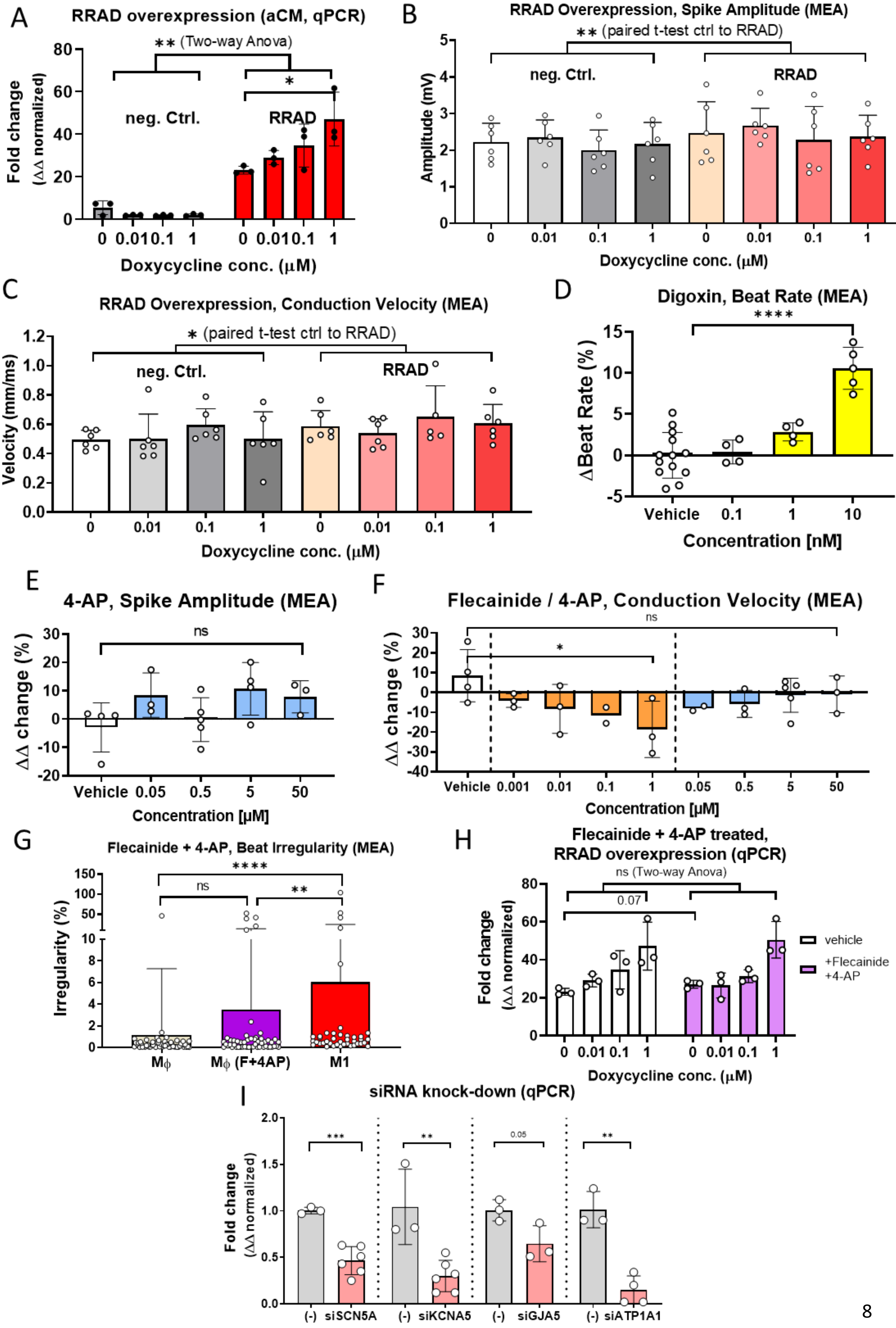
A
RRAD overexpression (aCM, qPCR)
** (Two-way Anova)
*
neg. Ctrl.
RRAD
Fold change (ΔΔ normalized)
0 0.01 0.1 1 0 0.01 0.1 1
Doxycycline conc. (µM)
B
RRAD Overexpression, Spike Amplitude (MEA)
** (paired t-test ctrl to RRAD)
neg. Ctrl.
RRAD
Amplitude (mV)
0 0.01 0.1 1 0 0.01 0.1 1
Doxycycline conc. (µM)
C
RRAD Overexpression, Conduction Velocity (MEA)
* (paired t-test ctrl to RRAD)
neg. Ctrl.
RRAD
Velocity (mm/ms)
0 0.01 0.1 1 0 0.01 0.1 1
Doxycycline conc. (µM)
D
Digoxin, Beat Rate (MEA)
****
ΔBeat Rate (%)
Vehicle 0.1 1 10
Concentration [nM]
E
4-AP, Spike Amplitude (MEA)
ns
ΔΔ change (%)
Vehicle 0.05 0.5 5 50
Concentration [µM]
F
Flecainide / 4-AP, Conduction Velocity (MEA)
ns
*
ΔΔ change (%)
Vehicle 0.001 0.01 0.1 1 0.05 0.5 5 50
Concentration [µM]
G
Flecainide + 4-AP, Beat Irregularity (MEA)
****
ns
**
Irregularity (%)
Mϕ
Mϕ (F+4AP)
M1
H
Flecainide + 4-AP treated,
RRAD overexpression (qPCR)
ns (Two-way Anova)
0.07
Fold change (ΔΔ normalized)
vehicle
+Flecainide +4-AP
0 0.01 0.1 1 0 0.01 0.1 1
Doxycycline conc. (µM)
I
siRNA knock-down (qPCR)
***
**
0.05
**
Fold change (ΔΔ normalized)
(-) siSCN5A
(-) siKCNA5
(-) siGJA5
(-) siATP1A1

Figure S5: Compound treatment identifies inhibition of Na+/K+ pump activity in aCM to be pro-arrhythmic, while upregulation of *RRAD* or inhibition of Nav1.5 or I_{kur} to not affect beat regularity

A) qPCR mRNA expression analysis for *RRAD,* in 2D aCM transduced with a doxycycline inducible RRAD or negative control lentiviral vector, normalized to GAPDH and ACTN housekeeping gene expression levels (n=3/N=1, One Way ANOVA intragroup, Two Way ANOVA intergroup). **B)** MEA recordings of spike amplitude of 2D aCM transfected with a doxycycline inducible RRAD or negative control vector (n=6/N=1, paired student t-test). **C)** MEA recordings of conduction velocity in aCM monolayers transduced with a doxycycline inducible RRAD or negative control lentiviral vector (n=6/N=1, paired student t-test). MEA recordings in 2D aCM cultures treated with **D)** digoxin, showing dose dependent increase in beat rate (n=13,4,4,5/N=1, One Way ANOVA), and **E)** spike amplitude (n=4,3,5,4,3/N=1, One Way ANOVA) and **F)** conduction velocity values of flecainide or 4-AP treated aCM (n=4,3,3,2,3,2,3,5,3/N=1, One Way ANOVA). **G)** MEA recordings of beat irregularity in 2D aCM+M1, and aCM+Mϕ coculture treated with combination of flecainide and 4-AP, showing no significant increase in beat irregularity in treated cultures (n=56,53,41/N=3, Kruskal-Wallis test). **H)** qPCR expression of *RRAD* in 2D aCM transduced with a doxycycline inducible RRAD or negative control vector and treated with flecainide and 4-AP, normalized to GAPDH and ACTN expression levels (n=3/N=1, Two Way ANOVA intergroup, unpaired student t-test 0 µM comparison). **I)** qPCR expression in 2D aCM treated with siRNA against *SCN5A, KCNA5*, *GJA5*, *ATP1A1* or negative control (-), normalized to GAPDH and (-) expression levels (n=3,6,3,6,3,3,3,4/N=3, unpaired student t-test). ns: not significant, *: P<0.05, **: P<0.01, ***: P<0.001, ****: P<0.0001

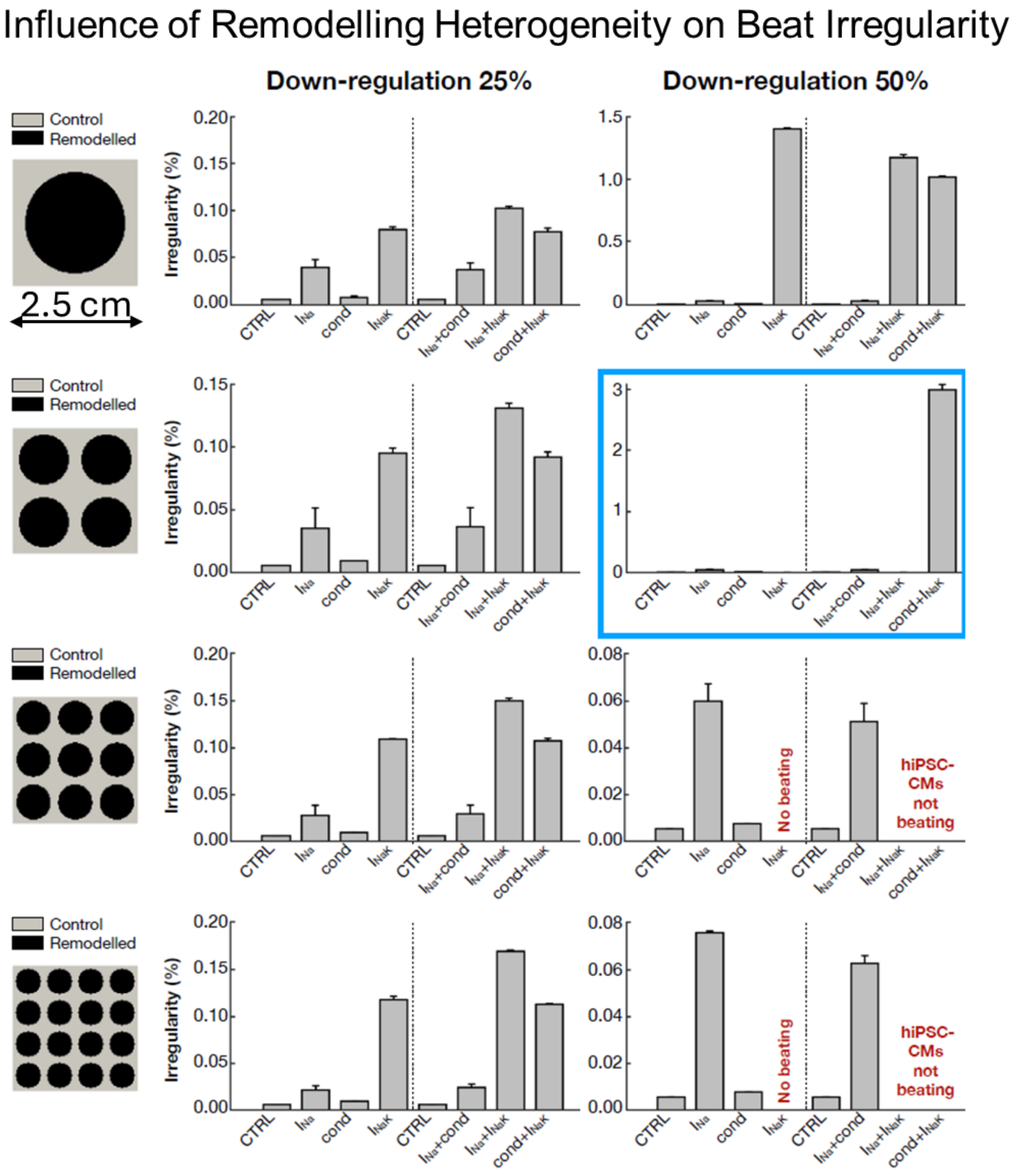

Figure S6: Degree of spatial remodeling complexity affects inducibility of beat irregularity by downregulation of electrophysiological parameters in an hiPSC-CM *in silico* model

Comparison of beating irregularity in an *in silico* hiPSC-CM tissue between control conditions (CTRL – no remodeling) and six scenarios of electrophysiological remodeling applied in four spatially heterogeneous configurations. Electrophysiological remodeling was applied to cover 50% of the whole tissue in circular patches of 1 cm radius (one circular patch), 0.5 cm radius (four circular patches), 0.34 cm radius (nine circular patches) and 0.25 cm radius (sixteen circular patches). For nine and sixteen circular patches, 50% I_{NaK} down-regulation prevented spontaneous beating of hiPSC-CMs. The blue box highlights the scenario included in Figure 6. Y-axis ranges automatically generated by simulation algorithms and vary between conditions tested.

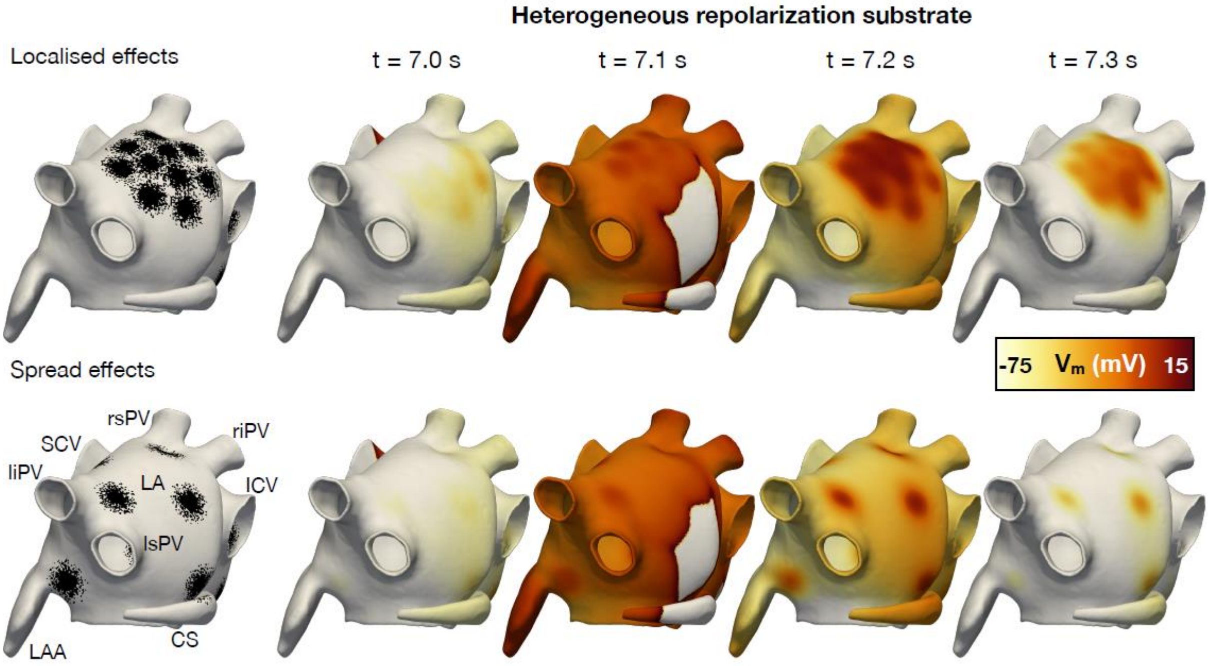

Figure S7: Heterogeneous repolarization substrate resulting from electrophysiological remodeling involved in inflammation

Consecutive snapshots of the transmembrane voltage (V_m) in two representative 3D atrial models, one with localized and one with spread patches of remodeling. Remodeled patches prolonged the action potential duration locally, creating heterogeneous repolarization. Abbreviations. LA: left atrium; LAA: LA appendage; SCV-ICV: superior and inferior cava vein; rs-ri-ls-li-PV: right superior, right inferior, left superior and left inferior pulmonary vein; CS: coronary sinus.

Table S1

Supplementary Table 2: List of Antibodies (ordered by mention)

Name	Target	Dilution	Supplier	Species	Reactivity
cTnT Reafinity conjugated FITC	cTnT	1:10 (Flow cytometry), 1:100 (IF)	Miltenyi	Human	Human
MLC2a Reafinity conjugated APC	MLC2a	1:10 (Flow cytometry)	Miltenyi	Human	Human
REA control FITC	-	1:10 (Flow cytometry)	Miltenyi	Human	-
REA control APC	-	1:10 (Flow cytometry)	Miltenyi	Human	-
Human COUP-TF II/NR2F2 Antibody	COUP-TF II	1:100	R&D Systems	Mouse	Human
(APC) AffiniPure F(ab')$_2$ Fragment Donkey Anti-Mouse IgG (H+L)	-	1:500	Jackson ImmunoResearch	Donkey	Mouse
Purified Mouse IgG2a, κ	-	1:100	BioLegend	Mouse	-
CD45 PE	CD45	1:20	BioLegend	Mouse	Human
CD11b APC	CD11b	1:20 (Flow cytometry)	BioLegend	Mouse	Human
CD14 FITC	CD14	1:20 (Flow cytometry)	BioLegend	Mouse	Human
IgG1-PE	-	1:160	BioLegend	Mouse	-
IgG1-APC	-	1:40 (Flow cytometry)	BioLegend	Mouse	-
IgG1-FITC	-	1:10 (Flow cytometry)	BioLegend	Mouse	-
Vimentin REAfinity™ conjugated FITC	Vimentin	1:50	Miltenyi	Human	Human
CX3CR1 (1H14L7)	CX3CR1	1:250	Invitrogen	Rabbit	Human
IgG (H+L) Alexa Fluor® 488	-	1:500	ThermoFisher Scientific	Goat	Human
IgG (H+L) Alexa Fluor® 594	-	1:200	ThermoFisher Scientific	Donkey	Rabbit
IgG2b Alexa Fluor® 647	-	1:200	ThermoFisher Scientific	Goat	Mouse
IgG (H+L) Alexa Fluor® 594	-	1:200	ThermoFisher Scientific	Donkey	Rabbit
Collagen I Polyclonal Antibody	Collagen I	1:100	ThermoFIsher Scientific	Rabbit	Human
Alpha smooth muscle actin [1a4] mab1420	Alpha smooth muscle actin	1:50	R&D Systems	Mouse	Human
NG2/MCSP PE mouse ab	NG2	1:100	R&D Systems	Mouse	Human

Table S2

Supplementary Table 2: qPCR Primers and corresponding sequences (ordered alphabetically)

Primer	Supplier	Sequence	Chromosome Location	Amplicon Length
ACTB	Bio-Rad		7:5568936-5569027	62
ATP1A1	Bio-Rad		1:116929977-116930085	79
COL1A1	Bio-Rad		17:48262555-48262698	114
GAPDH	Integrated DNA Technologies	Fw: TCC TCT GAC TTC AAC AGC GA<br>Rv: GGG TCT TAC TCC TTC GAG GC		
GJA5	Integrated DNA Technologies	Fw: AAT CAG TGC CTG GAG AAT GG<br>Rv: CGA ACC TGG ATG AAA CCT TC		
KCNA5	Integrated DNA Technologies	Fw: CGA GGA TGA GGG CTT CAT TA<br>Rv: CTG AAC TCA GGC AGG GTC TC		
MMP2	Bio-Rad		16:55516872-55516983	82
NR2F2	Integrated DNA Technologies	Fw: CCG AGT ACA GCT GCC TCA A<br>Rv: TTT TCC TGC AAG CTT TCC AC		
SCN5A	Bio-Rad		3:38628942-38629064	93
